# Concentration-Dependent Photocatalytic Upcycling of Poly(ethylene terephthalate) Plastic Waste

**DOI:** 10.1021/acsmaterialslett.3c01134

**Published:** 2023-10-16

**Authors:** Hongxing Kang, Audrey Washington, Matt D. Capobianco, Xingxu Yan, Vayle Vera Cruz, Melanie Weed, Jackie Johnson, Gonto Johns, Gary W. Brudvig, Xiaoqing Pan, Jing Gu

**Affiliations:** † Department of Chemistry and Biochemistry, San Diego State University, 5500 Campanile Drive, San Diego, California 92182, United States; ‡ Department of Chemistry and Yale Energy Sciences Institute, Yale University, New Haven, Connecticut 06520-8107, United States; § Department of Materials Science and Engineering, University of California, Irvine, Irvine, California 92697, United States; ∥ Department of Physics and Astronomy, University of California, Irvine, Irvine, California 92697, United States

## Abstract

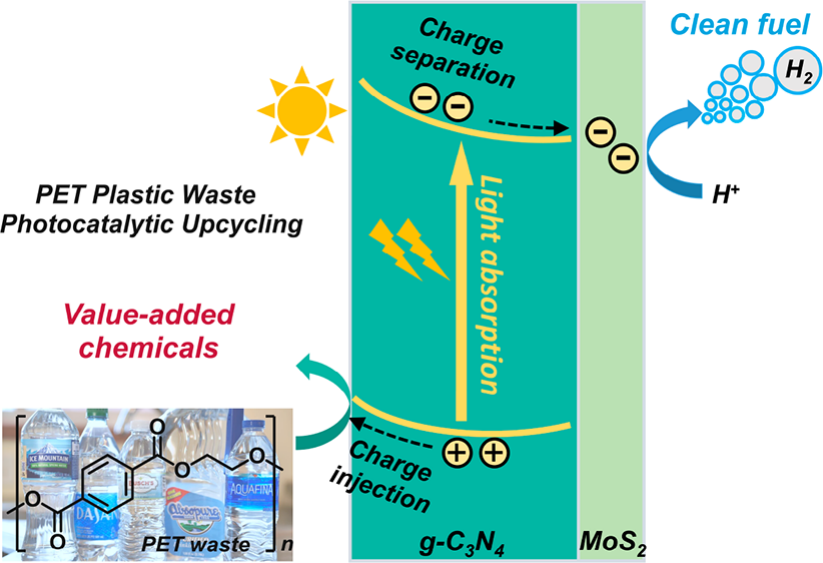

Photocatalytic plastic waste upcycling into value-added feedstock is a promising way to mitigate the environmental issues caused by the nondegradable nature of plastic waste. Here, we developed a MoS_2_/g-C_3_N_4_ photocatalyst that can efficiently upcycle poly(ethylene terephthalate) (PET) into valuable organic chemicals. Interestingly, the conversion mechanism is concentration-dependent. For instance, at a low ethylene glycol (EG) concentration (7.96 mM), acetate is the main product. Unexpectedly, the conversion of PET water bottle hydrolysate with only 7.96 mM ethylene glycol (EG) can produce a 4 times higher amount of acetate (704.59 nmol) than the conversion of 300 mM EG (174.50 nmol), while at a higher EG concentration (300 mM), formate is the dominant product. Herein, a 40 times higher EG concentration (300 mM compared to 7.96 mM) would produce only ∼3 times more formate (179 nmol compared to 51.86 nmol). In addition, under natural sunlight conditions, comparable amounts of liquid and gaseous products are produced when commercial PET plastics are employed. Overall, the photocatalytic PET conversion process is quite efficient under a low concentration of EG in PET hydrolysate, indicating the enormous potential of this photocatalysis strategy for real plastics upcycling.

Plastic materials have been increasingly manufactured and applied in ever-expanding industrial fields due to their low cost, durability, and processability since the appearance of the first synthetic polymer. In modern society, plastic materials with a wide range of applications have become ubiquitous and indispensable. Since the 1950s, more than 8300 million metric tons of plastics have been produced, and ∼80% of them are discarded and accumulated in the natural environment after use,^1,2^ resulting in severe environmental pollution to earth’s ecosystem.^3^ As one of the most used plastic materials, over 70 million tons of poly(ethylene terephthalate) (PET) are produced annually.^4^ Unlike natural polymers, PET wastes can accumulate on earth due to their relatively sluggish natural decomposition kinetics.^5,6^ To solve this problem, currently PET wastes are mainly recycled through mechanical and chemical methods. In the mechanical recycling process,^2^ clean high-purity PET wastes are mixed with virgin polymer in a transformation process to produce other end products. However, the recover-and-recycle rates for plastics are extremely low due to the inefficiency of mechanical recycling.^7^ On the other hand, PET wastes are commonly depolymerized into monomers in the chemical recycling process through hydrolysis,^8^ glycolysis,^9^ ammonolysis,^10^ and methanolysis.^11^ The biggest disadvantages of the chemical recycling process are its high cost and the need for extra sorting. In addition to these two most common methods, enzymes have been developed to digest high-crystalline PET, providing new opportunities for biobased plastic recycling.^12,13^ Although the aforementioned methods can recycle PET back into its application cycles, these processes still face limitations, such as harsh reaction conditions, low product yields, high cost, and difficulty in purifying products. Therefore, developing a sustainable method that is capable of upcycling PET wastes into value-added chemicals under mild conditions with minimum energy input and low cost is urgently needed.^14,15^

Photocatalysis has been recognized as an emerging and promising approach that harnesses inexhaustible solar energy to drive redox reactions for renewable energy conversions, such as biomass valorization and hydrogen production.^16−18^ Using biomass derived species as an example, those species (i.e., glycerol) can act as an electron donor and be oxidized in the photocatalysis process to value-added chemicals, while the protons (H^+^) are simultaneously reduced by photoexcited electrons to H_2_.^19^ Similar to biomass-derived compounds, PET wastes are ideal chemicals for photocatalytic conversion since their hydrolysis product, ethylene glycol (EG), is a common biomass-derived compound. Recently, several photocatalytic systems, such as CdS/CdO_*x*_ quantum dots,^20^ CN_*x*_/Ni_2_P,^21^ CN-CNTs-NiMo,^22^ and Ti–Fe_2_O_3_/Ni(OH)_*x*_,^23^ have been reported for PET photoreforming and producing value-added chemicals, such as formate, acetate, and hydrogen gas.^22^ However, the possible conversion mechanism (i.e., through radical intermediates) and the efficiency of photoreforming systems under low-concentration conditions in comparison with those under high-concentration conditions have been seldom discussed.

Herein, a MoS_2_/g-C_3_N_4_ photocatalyst, which is capable of efficiently oxidizing PET plastic waste to value-added chemicals while simultaneously reducing water into H_2_, was synthesized. The MoS_2_/g-C_3_N_4_ photocatalyst displays desirable performance for the upcycling of PET plastic waste (commercial PET powder and water bottle) by oxidizing the PET monomer, EG, into value-added liquids and gas fuels while reducing water into H_2_. In addition, three parallel reaction pathways are identified, where, in **Pathway 1**, the formation of ·CH_3_ radicals leads to the formation of CH_4_. In **Pathway 2**, H_2_ and CO were identified as the main products, which indicates that the H_2_ generated is not solely contributed by water reduction. Lastly, in **Pathway 3**, acyl radicals are oxidized to glycolate, which is sequentially oxidized to glyoxylate, oxalate, and eventually to formate. The experimental results indicate that, at high EG concentration (300 mM), **Pathway 3** is dominant; however, at low EG concentration (7.96 mM), **Pathway 1** is presiding. Finally, sunlight and real-world PET plastics were directly employed to explore the practical application potential of this system.

**Figure 1:**
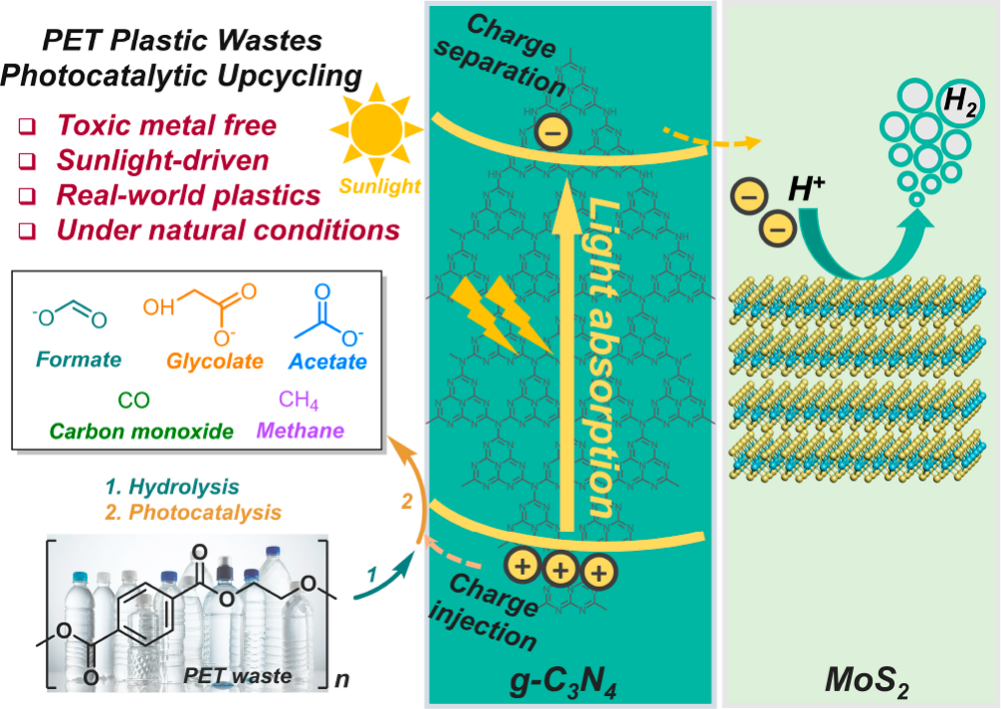
Schematic diagram of photocatalytic PET waste upcycling over the MoS_2_/g-C_3_N_4_ photocatalyst.

*MoS_2_/g-C_3_N_4_ Synthesis and Structural Characterizations*. In this work, graphitic carbon nitride (g-C_3_N_4_) was prepared from thermal polymerization of urea under an N_2_ atmosphere, while 1T-MoS_2_ was synthesized via a hydrothermal reaction following the previous work.^24^ The MoS_2_/g-C_3_N_4_ photocatalyst was fabricated by mixing the as-synthesized 1T-MoS_2_ and g-C_3_N_4_ under 300 °C (Figure S1), following slightly modified literature procedures (see the experimental section in the Supporting Information for details).^25^

The structures of the synthesized photocatalysts were analyzed by powder X-ray diffraction (XRD, Figure 2). Two characteristic diffraction peaks of g-C_3_N_4_, at approximately 2θ = 12.9 and 2θ = 27.5°, ascribed to the (100) and (002) crystal planes with equivalent *d*-spacings of 6.850 and 3.245 Å, respectively, were identified.^26^ After incorporating MoS_2_ catalysts, the (100) and (002) diffraction peaks of MoS_2_/g-C_3_N_4_ are well retained, indicating that the incorporated MoS_2_ does not change the lattice spacing of g-C_3_N_4_. At the same time, due to the low concentration of MoS_2_ (1 wt %), hardly any MoS_2_ diffraction peaks can be identified. However, it is clear that 1T-MoS_2_ was successfully synthesized supported by Raman spectra and Fourier transform infrared (FTIR) (Figure S2). For instance, Raman peaks displaying at 146, 235, and 334 cm^–1^, which are corresponding to the J_1_, J_2_, and J_3_ characteristic vibration modes of 1T-MoS_2_, can be confidently assigned (Figure S2a).^27^

**Figure 2:**
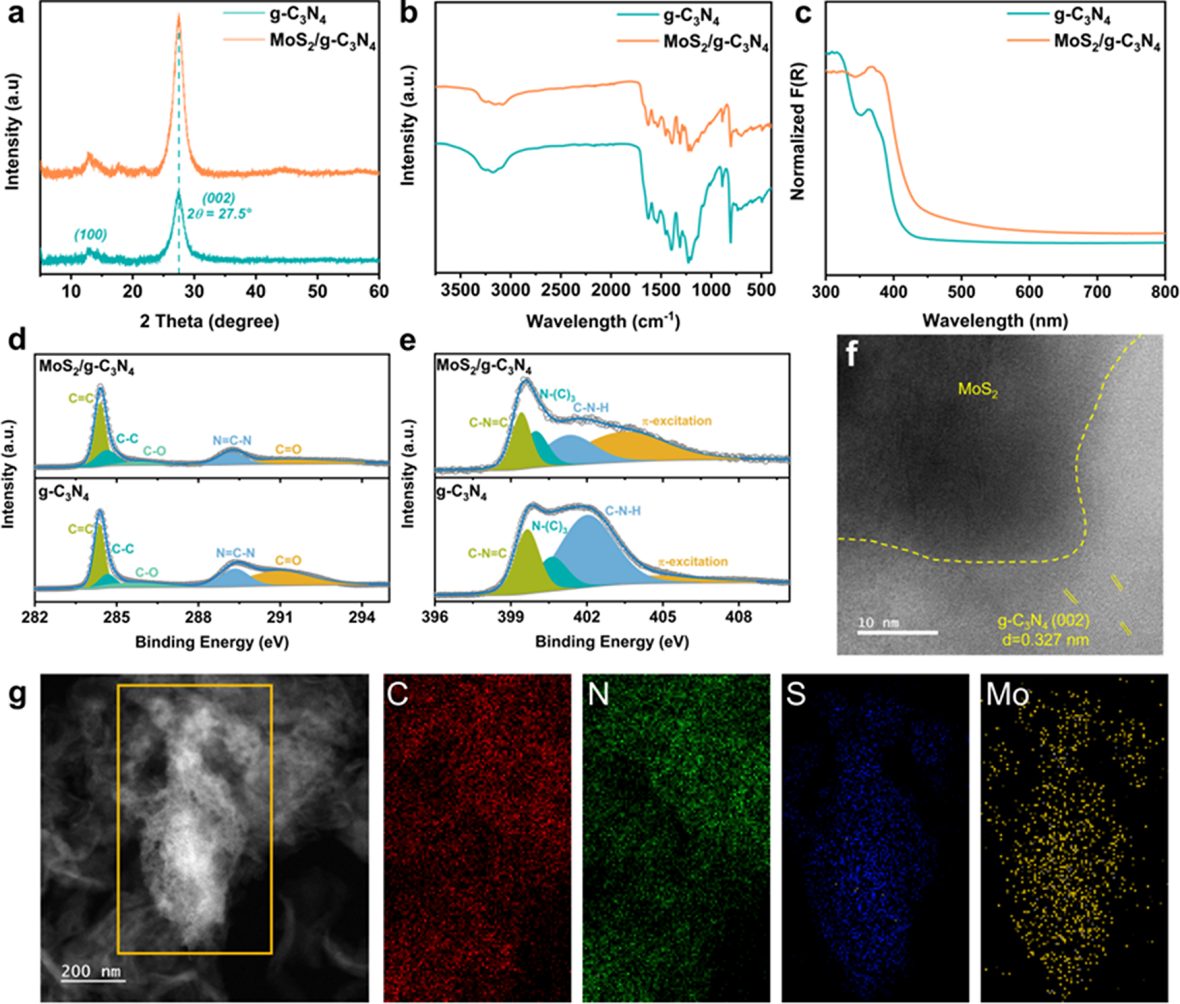
Structural characterizations of g-C_3_N_4_ and MoS_2_/g-C_3_N_4_ photocatalysts. (a) XRD patterns, (b) FTIR spectra, (c) diffuse-reflectance UV–vis spectra, XPS spectra of (d) C 1s and (e) N 1s, (f) STEM image, and (g) STEM-EDX maps of MoS_2_/g-C_3_N_4_.

In addition, FTIR spectroscopy was carried out to reveal the structures of g-C_3_N_4_ and MoS_2_/g-C_3_N_4_ (Figure 2b). For g-C_3_N_4_, the characteristic breathing mode of tri-s-triazine and the deformation mode of N–H in amino groups are observed at ∼806 and 889 cm^–1^, respectively.^28^ Additionally, stronger peaks are observed at approximately 1227, 1312, and 1397 cm^–1^, corresponding to the aromatic C–N stretching vibrations. Similarly, C=N vibrations are observed at 1538 and 1627 cm^–1^.^29,30^ Further, the broad bands at 3074–3325 cm^–1^ are assigned to the possible existence of N–H bonds from the uncondensed amine groups^31^ and O–H vibrations from absorbed H_2_O.^32^ In summary, FTIR spectra of MoS_2_/g-C_3_N_4_ and g-C_3_N_4_ show similar characteristic peaks, suggesting that the incorporation of MoS_2_ does not influence the bulk properties of g-C_3_N_4_. This result agrees well with the XRD results.

The optical properties of the samples were characterized by UV–vis diffusive reflectance spectroscopy (DRS). As shown in Figure 2c, the absorption edge of pristine g-C_3_N_4_ is located at 450 nm, while MoS_2_/g-C_3_N_4_ displayed a clearly red-shifted absorption edge (∼500 nm) and a slightly enhanced absorption at 350 nm. The shift of the absorption edge might be explained by the heterojunction structure formed between MoS_2_ and g-C_3_N_4_, which promotes visible light absorption and charge carrier generation.^33^ In addition, the slightly enhanced absorption is in good agreement with the observed color change (light yellow to gray) (Figure S3). In addition, the Tauc plot (Figure S4a) was derived from the corresponding UV–vis DRS to determine the band gap; MoS_2_/g-C_3_N_4_ (2.61 eV) displayed a narrower band gap than g-C_3_N_4_ (2.79 eV). The positive slopes of MoS_2_/g-C_3_N_4_ and g-C_3_N_4_ in the Mott–Schottky (M–S) curves (Figure S4b) confirm its n-type semiconductor characteristic.

The photoluminescence (PL) spectra are employed to investigate the photogenerated carrier transfer and recombination process during photocatalysis. As shown in Figure S4c, the PL spectrum of the g-C_3_N_4_ spectrum shows a strong emission centered at ∼440 nm. The formation of MoS_2_/g-C_3_N_4_ heterostructure results in a decrease in PL intensity, indicating a suppressed photogenerated carrier recombination.^34^ Furthermore, electrochemical impedance spectroscopy (EIS) was carried out to evaluate the changes in the charge transfer resistance. Compared to pristine g-C_3_N_4_, MoS_2_/g-C_3_N_4_ displays a smaller semicircle radius (Figure S4d), which implies a faster interfacial charge transfer kinetics.^35^

Furthermore, the chemical composition and elemental state of the as-synthesized MoS_2_/g-C_3_N_4_ were analyzed by X-ray photoelectron spectroscopy (XPS). The survey spectra reveal the presence of C, N, and O elements (Figure S5a). Notably, the high-resolution C 1s and N 1s spectra of g-C_3_N_4_ and MoS_2_/g-C_3_N_4_ were slightly shifted from the literature values, probably due to the charging effect induced by the low conductivity of g-C_3_N_4_.^36,37^ The deconvoluted C 1s spectra of g-C_3_N_4_ exhibit five peaks (Figure 2d) with lower binding energy peaks at 284.40 and 284.60 eV, separately assigned to C=C and C–C.^38−40^ These peaks can be originated from adventitious carbon, defects in g-C_3_N_4_, or carbon fiber paper (CFP).^41^ In addition, the peak at 285.82 eV is assigned to C–O^42^ on the g-C_3_N_4_ surface, while the peak at 289.24 eV is assigned to the sp^2^-bonded N=C–N in the triazine ring.^43^ The high binding energy peak at 291.22 eV is attributed to the existence of C=O, possibly originating from the incomplete polycondensation of the urea precursor.^44^ The N 1s (Figure 2e) spectra of g-C_3_N_4_ show the existence of four peaks at 399.41, 399.97, 401.30, and 403.43 eV, corresponding to the presence of C–N=C, N–(C)_3_, C–N–H side groups, and π-excitation, respectively.^30^ The high-resolution C 1s and N 1s spectra of g-C_3_N_4_ and MoS_2_/g-C_3_N_4_ are nearly identical, suggesting that the electronic properties of g-C_3_N_4_ are well-retained after adding MoS_2_. Compared with pristine g-C_3_N_4_, the Mo peak was identified in the Mo 3d spectra (Figure S5b), further confirming the presence of MoS_2_ in the MoS_2_/g-C_3_N_4_ composite. The absence of the S element probably stems from the small quantity (∼1 wt %) of MoS_2_ in the MoS_2_/g-C_3_N_4_ composite.

To further confirm the structure of the MoS_2_/g-C_3_N_4_ composite, scanning electron transmission microscopy (STEM) was conducted to reveal the successful formation of a heterojunction between MoS_2_ and g-C_3_N_4_. In Figure 2f, the interlayer distances of 0.327 and 0.620 nm, corresponding to the presence of the (002) plane of g-C_3_N_4_ and the (002) plane of MoS_2_ (Figure S6), respectively, can be identified. Herein, the MoS_2_ interlayer distance indicates that MoS_2_ exists as a few-layer MoS_2_ film, corresponding well with the few-layer morphology of the 1T-MoS_2_ precursor.^45^ The corresponding elemental mapping in Figures 2g and S7 validates the uniform distribution of C, N, S, and Mo elements. Lastly, the SEM images of MoS_2_/g-C_3_N_4_ and g-C_3_N_4_ are displayed in Figures S8 and S9.

*PET Waste Pretreatment*. To obtain desirable photocatalytic performance, the commercial PET powder and a PET water bottle (brand: Aquafina) were pretreated in 2 M KOH to depolymerize PET into monomers (see Supporting Information for details) via the base-catalyzed polyester hydrolysis reaction (Figure S10). The hydrolysis product was identified by ^1^H NMR and LC-MS.

The major chemicals identified from the Aquafina water bottle are similar to the ones derived from PET powder. For commercial PET powder, as shown in Figures S11a and S12a, terephthalate (a, *m*/*z* = 165) and ethylene glycol (g, 174.60 mM) were identified as the main products after pretreatment, together with a minor product, isophthalate (*m*/*z* = 121), an isomer of terephthalate, by LC-MS and ^1^H NMR. In comparison, the major hydrolysis products of the Aquafina PET water bottle were terephthalate (a) and ethylene glycol (g) (7.96 mM) with no isomers recognized (Figure S11b) by ^1^H NMR. However, more minor peaks were presented in the LC-MS spectrum, possibly due to the presence of extra additives in the commercial water bottles (Figure S12b).

*Photocatalytic Hydrogen Evolution Reaction (HER)*. As-synthesized photocatalyst was initially evaluated via photocatalytic HER, which was conducted in a gastight vial with triethanolamine (TEOA) as the sacrificial electron donor in DI H_2_O (see Supporting Information for details). The gaseous products, such as H_2_, were analyzed by gas chromatography (GC).

Photocatalytic HER performance was employed to optimize the synthesis method of the photocatalyst. Compared to the physically mixed MoS_2_/g-C_3_N_4_, the MoS_2_/g-C_3_N_4_ after annealing exhibited a significantly increased HER performance with up to a 31.08 μmol g^–1^ H_2_ yield (Table S1) with a 0.5 wt % MoS_2_ loading amount. In contrast, unannealed MoS_2_/g-C_3_N_4_ can only achieve only 1.01 μmol g^–1^ H_2_ yield, 30 times smaller than that of the annealed MoS_2_/g-C_3_N_4_. The significantly enhanced HER performance is ascribed to the successful formation of a heterojunction between MoS_2_ and g-C_3_N_4_, which has been known to facilitate the photoinduced electron transfer from g-C_3_N_4_ to MoS_2_.^46^ Furthermore, different MoS_2_ loading amounts (0.5, 1, and 2 wt %) were studied, where the optimal MoS_2_ loading amount is identified to be 1 wt % (Table S2). A lower MoS_2_ loading amount (0.5 wt %) is not sufficient for photoexcited electron extraction, while a higher amount of MoS_2_ (2 wt %) might decrease the light absorption of g-C_3_N_4_. From then on, 1 wt % MoS_2_/g-C_3_N_4_ was further employed and denoted as MoS_2_/g-C_3_N_4_ if no further notifications. In summary, the H_2_ production rate (Table S2) decreased with the reaction time, which could be attributed to the consumption of the electron donor (TEOA).

To obtain a desirable PET upcycling performance, PET requires a pretreatment under alkaline conditions to depolymerize it to monomers: EG and TPA prior to photocatalysis. Therefore, photocatalytic HER performance over MoS_2_/g-C_3_N_4_ was further investigated under near-neutral H_2_O, 1 M KOH, and 2 M KOH to reveal the potential benefit of the alkaline solution. As shown in Figure 3a, H_2_ yield, from 35.73 μmol g^–1^ (in H_2_O) to 49.80 μmol g^–1^ (in 2 M KOH), was significantly enhanced with an increased pH over 2 h of photocatalysis, indicating the good tolerance of MoS_2_/g-C_3_N_4_ photocatalyst under a strong alkaline condition.

**Figure 3:**
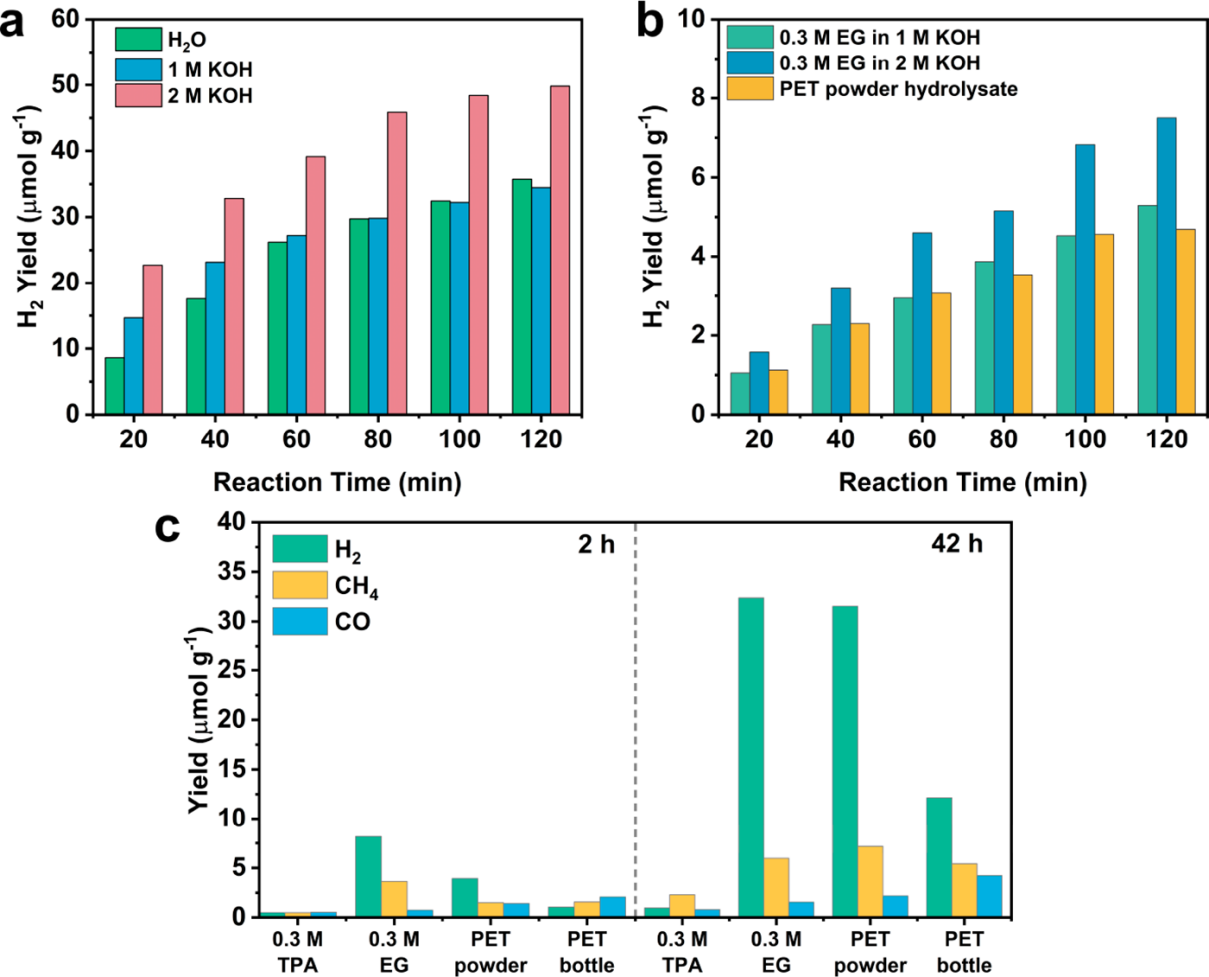
Photocatalytic performance with different conditions over MoS_2_/g-C_3_N_4_ photocatalyst. (a) pH effect on photocatalytic H_2_ yield; TEOA was used as an electron donor. (b) Photocatalytic H_2_ yield; 0.3 M EG and PET powder hydrolysates were used as electron donors, respectively. (c) Comparison of gaseous products for short-term (2 h) and long-term (42 h) photocatalysis with various substrates.

*Photocatalytic Substrate Oxidation*. After optimizing catalysts and photocatalytic conditions, 0.3 M EG was employed as the electron donor instead of TEOA to study the photocatalytic HER activity while mimicking the conditions for PET monomer oxidation. Similarly, as in the case of TEOA, photocatalytic H_2_ evolution performance was enhanced with an increased pH (2 M KOH (7.50 μmol g^–1^ for 2 h) compared to 1 M KOH (5.29 μmol g^–1^ for 2 h)) (Figure 3b). Moreover, when commercial PET powder (hydrolysis in 2 M KOH) was used (Figure 3b), a comparable H_2_ evolution yield of 4.69 μmol g^–1^ was achieved. These results suggest PET plastic could be upcycled through base-catalyzed hydrolysis and subsequent photocatalysis steps over MoS_2_/g-C_3_N_4_.

To further explore the mechanism of photocatalytic PET waste conversion, 2 h photocatalysis experiments were conducted. As shown in Table S3, compared to the negligible amount of H_2_ in the air (0.21 μmol g^–1^), the presence of light and MoS_2_ catalyst is necessary for photocatalytic PET powder hydrolysate upcycling. More specifically, g-C_3_N_4_ is capable of photocatalytically converting H_2_O into H_2_, but the H_2_ amount generated (1.26 μmol g^–1^) is only one-third of the MoS_2_/g-C_3_N_4_ (3.93 μmol g^–1^) after 2 h of photoirradiation. Additionally, when 0.3 M TPA was applied as an electron donor, the H_2_ yield (0.47 μmol g^–1^) resembles that of no catalyst (0.40 μmol g^–1^) and no light (0.58 μmol g^–1^) conditions, suggesting TPA cannot act as a sacrificial reagent. This experiment further confirms the fact that only monomer EG can be oxidized in the photocatalytic PET upcycling process. Moreover, when a PET water bottle hydrolysate was employed as the substrate, the H_2_ yield reaches 1.05 μmol g^–1^, suggesting the real-world application potential of this photocatalytic system. In addition, besides H_2_, CH_4_ and CO were identified as gaseous products with 0.3 M EG, PET powder, and PET water bottle hydrolysates as substrates, respectively. Meanwhile, liquid products, formate, glycolate, and acetate, were identified by ^1^H NMR (Figures S14 and S15).

*Long-Term Photocatalytic Reaction*. In addition, to obtain enough gas and liquid products for a more accurate quantitative analysis, the products were analyzed after a 42 h photocatalytic reaction. After the long-term reaction, H_2_, CH_4_, and CO were confirmed as the main gaseous products by GC (Figure S16). More specifically, 31.53 and 12.13 μmol g^–1^ of H_2_ were produced from PET powder and a PET water bottle, respectively. Additionally, CH_4_ and CO yields are significantly elevated in comparison with the short-term experiments (Figure 3c). Notably, the H_2_ yield from a PET water bottle hydrolysate is lower than that of PET powder. In addition, negligible liquid and gaseous products were found when 0.3 M TPA was used as the substrate (Figure S19b), further confirming that TPA cannot be oxidized in the photocatalytic reaction. It is noticeable that the total yield of gaseous products from the PET water bottle hydrolysate (EG, 7.96 mM) is only 2 times lower than that of PET powder hydrolysate (EG, 174.60 mM) and 300 mM pure EG (Table S3), which suggests that the efficiency of this MoS_2_/g-C_3_N_4_ photocatalysis system for upcycling PET plastics to gas fuels is less limited by the concentration of the substrate. Meanwhile, this MoS_2_/g-C_3_N_4_ photocatalyst also demonstrates reasonable stability after a seven-cycle 2 h photocatalysis experiment where an almost linear cumulative H_2_ yield was observed (Figure S20). The FTIR spectrum (Figure S21) of MoS_2_/g-C_3_N_4_ postphotocatalysis displays identical features as prior to photocatalysis, suggesting that the photocatalyst structure remains intact. XPS spectra (Figure S22) further confirmed the unchanged surface properties of MoS_2_/g-C_3_N_4_ after the photocatalysis reaction, especially the deconvolution of N 1s (Figure S22c) spectrum exhibiting the same N species as prior to reaction. These results indicate that the as-prepared photocatalyst could be recycled and reused. To prove the robustness of the catalyst, lactic acid, which is the only hydrolysis product of another polyester plastic: poly(lactic acid) (PLA), was further employed. A comparable H_2_ yield (Table S4) was observed, while formate and acetate (Figure S23a) were identified as liquid products in 2 M KOH. This result indicates the potential application of the MoS_2_/g-C_3_N_4_ for other polyester plastics upcycling.

*Concentration-Dependent Photocatalytic Oxidation Mechanism of Ethylene Glycol*. As confirmed by the control experiments, the PET photocatalytic upcycling products are generated from EG oxidation. In the photoreforming of oxygenated substrates, two routes are usually proposed:^47^ (i) direct oxidation of substrates by holes on the surface of the photocatalyst or (ii) indirect oxidation of substances with hydroxyl (·OH) radicals generated by reacting the photocatalyst with water. To investigate the possibility of ·OH formation, we implemented photocatalytic upcycling of TPA as the ·OH scavenger. As shown in Figure S24a, TPA is prone to combine with ·OH to form 2-hydroxyterephthaclic (TPA–OH), which exhibits a characteristic photoluminescence (PL) peak at ∼430 nm under 320 nm excitation. After 42 h of photocatalytic upcycling of TPA, the TPA–OH PL peak is negligible (Figure S24b), which indicates that ·OH plays a minimal role in the EG photocatalytic oxidation reaction. Thus, herein the EG monomer photooxidation undergoes direct oxidation by photogenerated holes.

Based on the photocatalysis gas and liquid products, three C_1_ species and two C_2_ species are formed after 42 h of photoirradiation (Figure 4 and Table 1): CO (1.54, 5.98 μmol g^–1^), methane (5.98 μmol g^–1^), formate (179.07 nmol), glycolate (52.39 nmol), and acetate (174.50 nmol), respectively. We conclude that at least three parallel reaction pathways for EG photooxidation exist considering CH_4_ is the decarboxylation product of acetate (Scheme 1) according to previous literature.^18,21,48^ Herein, **Pathway 1** proceeds via dehydration of EG and further tautomerization to acetaldehyde,^49^ which undergoes a two-electron oxidation to form acetic acid. Further, acetic acid can undergo a photocatalytic decarboxylation (photo-Kolbe reaction) to form ·CH_3_ radicals,^50^ which will be terminated by the absorbed hydrogen on MoS_2_, generating CH_4_. In **Pathway 2**, EG is oxidized to form glycolaldehyde. Subsequently, under the photoirradiation condition, an acyl radical undergoes a decarbonylation reaction to deliver formaldehyde and CO.^19^ At the same time, acyl radicals can be oxidized to glycolate, which sequentially is oxidized to glyoxylate, oxalate, and eventually formate, as shown in **Pathway 3**. The control experiment for 300 mM glycolic acid photooxidation (Table S4) confirms the feasibility of converting glycolate into formate (Figure S23b), validating the rationality of **Pathway 3.**

**Figure 4:**
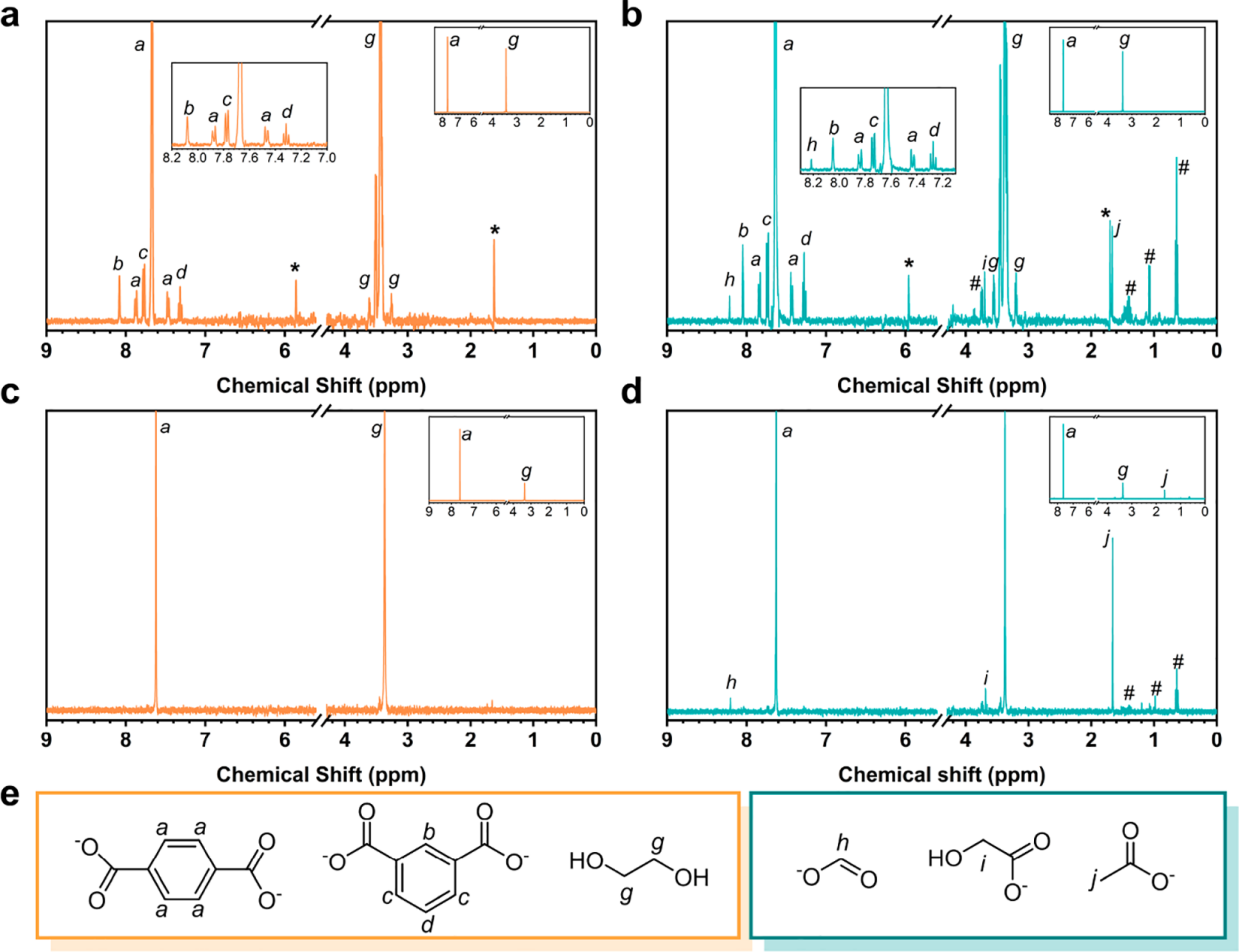
^1^H NMR spectra of PET powder hydrolysate and PET water bottle hydrolysate before (a,c, respectively) and after (b,d, respectively) 42 h of photocatalysis. (e) Corresponding peak assignments and chemical structures. (*) are seen from control experiments. (#) refers to unidentified products. Photocatalysis conditions: 2 mg of MoS_2_/g-C_3_N_4_, 1.25 mL of PET hydrolysate, 1 sun of xenon lamp light intensity.

**Table 1:** Liquid Product Yields after 42 h of Photocatalysis^a^

		Substrates photocatalysis		
		300 mM EG	PET powder hydrolysate (174.60 mM EG)	PET water bottle hydrolysate (7.96 mM EG)
	Formate	179.07	155.22	51.86
Products quantity (nmol)	Glycolate	52.39	53.50	39.54
	Acetate	174.50	340.15	704.59

a These values were calculated according to the ^1^H NMR peak area integration based on the calibration curves of ethylene glycol, formate, glycolate, and acetate (Figure S18).

**Scheme 1:**
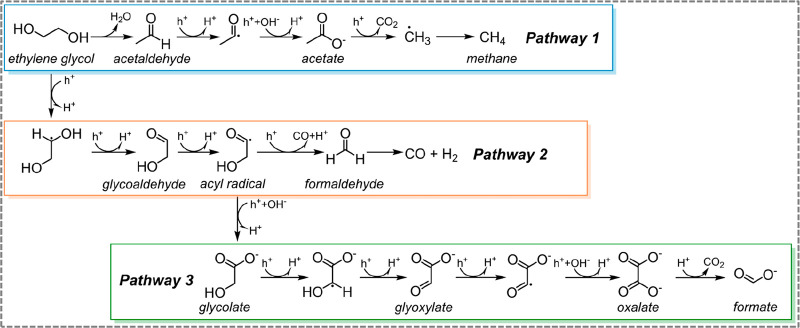
Proposed Ethylene Glycol (EG) Photocatalytic Reaction Pathways

Interestingly, as shown in Table 1, the selectivity for liquid products is concentration-dependent. Herein, the control sample (300 mM EG) is compared with PET powder hydrolysate (174.6 mM) and PET bottle hydrolysate (7.96 mM), in which the PET powder hydrolysate has a half time and PET bottle hydrolysate has ∼40 times smaller EG concentrations than that of the control sample. More specially, the acetate yield is elevated from 174.50 nmol (300 mM EG) to 340.15 nmol (PET powder hydrolysate) to 704.59 nmol (PET water bottle hydrolysate), while the formate yield is decreased from 179.07 nmol (300 mM EG) to 155.22 nmol (PET powder hydrolysate) to 51.86 nmol (PET water bottle hydrolysate) with the decreased EG concentration, suggesting that **Pathway 1** is the dominant route when the concentration of EG is low, while **Pathway 3** gains ascendancy under higher EG concentration. Moreover, it is worth mentioning that although the EG concentration is ∼40 times smaller in the PET water bottle hydrolysate (7.96 mM EG) than in the control sample (300 mM EG), the yield of formate is only 3 times smaller (51.86 nmol compared to 179.07 nmol). This result further indicates that this MoS_2_/g-C_3_N_4_ photocatalyst can achieve high photocatalytic efficiency for upcycling PET plastics to valuable products in a wide window of substrate concentration.

*Application in Real-World Environments*. Lastly, to investigate the potential of photocatalytic upcycling of PET waste under real-world conditions, the reaction setup was moved from the laboratory to the natural sunlight and atmospheric temperature condition (see inset in Figure 5). As shown in Figure 5, the natural sunlight intensity was measured to be around 0.60 to 0.75 sun, which is lower than the xenon lamp intensity (1 sun) used in the lab. For commercial PET powder and the PET water bottle hydrolysates, after 2 h of sun irradiation, the H_2_ yield was 5.12 and 0.95 μmol g^–1^, respectively. In addition, similar to the lab conditions, CH_4_ and CO were detected (Table 2). Moreover, formate (120.77 nmol), glycolate (53.86 nmol), and acetate (135.52 nmol) were observed as liquid products for the PET powder hydrolysate, while only acetate (46.85 nmol) was detected for the PET bottle hydrolysate (Table S5). These results from real-world conditions indicate that PET plastic waste can be upcycled to value-added organics driven by sunlight in the natural environment. Moreover, this system is expected to work more efficiently in areas where sunlight is more intense (i.e., places that are close to the equator)

**Figure 5:**
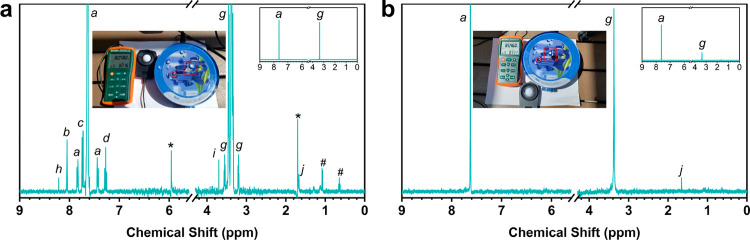
^1^H NMR of PET powder (a) and PET water bottle hydrolysates (b) after 2 h of photocatalysis under sunlight.

**Table 2:** Yields of Gaseous Products from PET Powder Hydrolysate and PET Water Bottle Hydrolysate after 2 h of Photocatalysis Driven by Sunlight over MoS_2_/g-C_3_N_4_

Catalyst	Substrate	Sunlight intensity (sun)	Time (h)	H_2_ yield (μmol g^–1^)	CH_4_ yield (μmol g^–1^)	CO yield (μmol g^–1^)
MoS_2_/g-C_3_N_4_	PET powder hydrolysate	0.67	2	5.12	1.86	2.55
	PET water bottle hydrolysate	0.71	2	0.95	1.58	0.77

*Photocatalytic Upcycling of Plastic Mixtures*. Photocatalytic plastic mixture upcycling was further conducted to evaluate the performance of MoS_2_/g-C_3_N_4_ for plastic mixture conversion. As shown in Figure 6a, after 42 h of photocatalysis, H_2_, CH_4_, and CO were observed as gaseous products. Compared to the pure PET plastic hydrolysates, in which H_2_ was observed as the main gaseous product, CH_4_ becomes the dominant gaseous product in PET + low-density polyethylene (LDPE) and PET + polystyrene (PS) mixtures. In addition, the CH_4_ yield is comparable to that of H_2_ in the mixture of PET + PS + LDPE. The enhanced CH_4_ yield with plastic mixtures as substrates could be ascribed to the decomposition of LDPE and PS under photoirradiation conditions.

**Figure 6:**
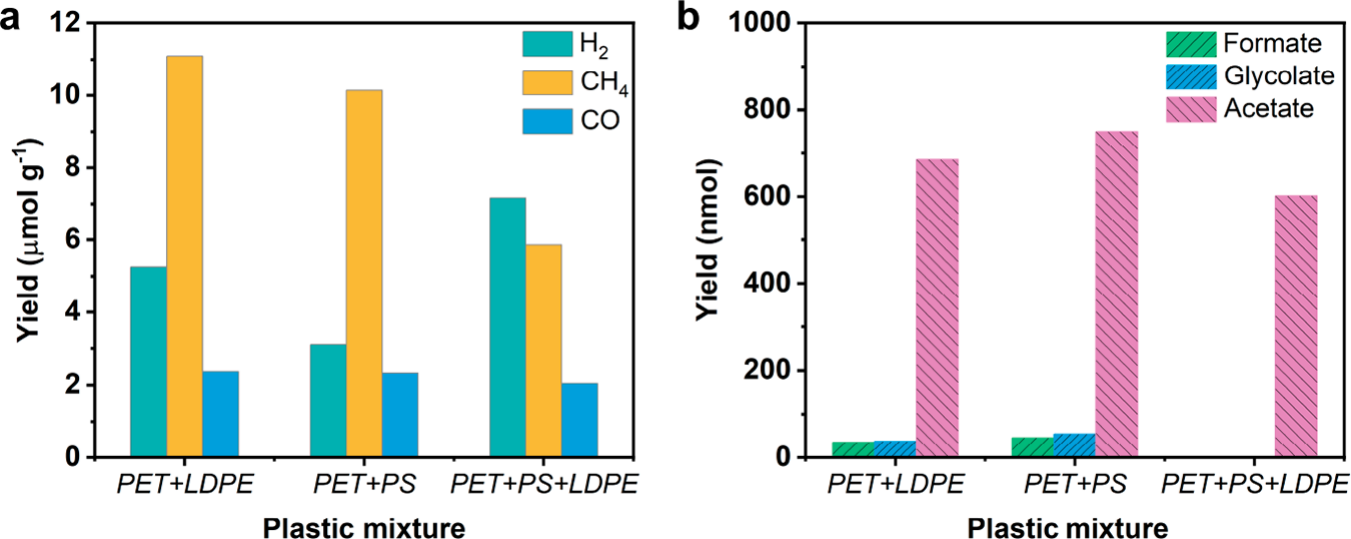
(a) Gaseous product and (b) liquid product yields from different plastic mixtures after 42 h of photocatalysis. Photocatalysis conditions: 2 mg of MoS_2_/g-C_3_N_4_, 1.25 mL of PET plastic mixture hydrolysate, and 1 sun of xenon lamp light intensity.

Furthermore, the liquid products were quantified based on the ^1^H NMR calibration curves. Herein, acetate was identified as the main liquid product in these plastic mixtures (Figures 6b and S25). Compared to the 300 mM EG and PET powder hydrolysate (174.60 mM), this result further supports that the liquid product distribution in the EGOR over MoS_2_/g-C_3_N_4_ photocatalyst is concentration-dependent.

In this work, we reported MoS_2_/g-C_3_N_4_ photocatalysts for photocatalytic PET plastic waste upcycling. The formation of a MoS_2_ and g-C_3_N_4_ heterojunction facilitates photogenerated charge separation, enhancing the catalytic activities of the hybridized photocatalyst. In detail, MoS_2_/g-C_3_N_4_ exhibits excellent H_2_ production yield when TEOA, commercial PET powder (31.53 μmol g^–1^), and a real PET water bottle (12.13 μmol g^–1^) were used as sacrificial reagent. Meanwhile, a comprehensive EG photooxidation mechanism was proposed according to experimental results, which shows that even the PET water bottle hydrolysate (7.69 mM) has a EG concentration ∼40 times smaller than the control EG (300 mM), its acetate yield is 4 times higher than control EG. Since the concentration of monomers in real-world polyester plastic hydrolysate is usually low, this result indicates the feasibility and great potential of the photocatalysis strategy in plastics upcycling. Lastly, the practical application of this photocatalysis system (sunlight and real PET bottle) was further explored, where a similar distribution and the same order of magnitude of gaseous and liquid products were yielded under natural conditions.

## Supporting Information Available

Tables containing all photocatalytic experiment data; Raman spectroscopy; Fourier transform infrared spectroscopy; photoluminescence spectroscopy; electrochemical impedance spectroscopy; additional X-ray photoelectron spectroscopy, scanning electron transmission microscopy, scanning electron microscopy, and energy dispersive X-ray spectroscopy; liquid chromatography–mass spectroscopy; gas chromatography; ^1^H nuclear magnetic resonance (PDF)
