# Supplementary material for: Concentration-Dependent Photocatalytic Upcycling of Poly(ethylene terephthalate) Plastic Waste
Source: ACS Mater Lett. 2023 Oct 16;5(11):3032–41. doi: 10.1021/acsmaterialslett.3c01134 (PMC10630977; doi:10.1021/acsmaterialslett.3c01134)
Supplement: Supplementary file 1 — tz3c01134_si_001.pdf [file tz3c01134_si_001.pdf]

Supporting Information

Concentration-Dependent Photocatalytic Upcycling of Poly(ethylene terephthalate) Plastic Waste

*Hongxing Kang[a], Audrey Washington[a], Matt D. Capobianco[b], Xingxu Yan[c], Vayle Vera Cruz[a], Melanie Weed[a], Jackie Johnson[a], Gonto Johns III[a], Gary W. Brudvig[b], Xiaoqing Pan[c,d], Jing Gu[a],**

[a] Department of Chemistry and Biochemistry, San Diego State University, 5500 Campanile Drive San Diego, CA 92182, USA

[b] Department of Chemistry and Yale Energy Sciences Institute, Yale University, New Haven, Connecticut 06520-8107, United States

[c] Department of Materials Science and Engineering, University of California, Irvine, Irvine, CA 92697, USA

[d] Department of Physics and Astronomy, University of California, Irvine, Irvine, CA 92697, USA

Experimental Section

Chemicals

Urea (Certified ACS, Fisher), Ammonium heptamolybdate tetrahydrate [$(NH_4)_6Mo_7O_{24}\cdot 6H_2O$, 99% Alfa Aesar] and thioacetamide (CH_3CSNH_2, 99+% Acros Organics) were used without further purification. Poly(ethylene glycol) terephthalate (PET), low density polyethylene (LDPE), and polystyrene (PS) were purchased from Goodfellow.

Synthesis of g-C_3N_4

10 g urea was put into a crucible with a cover, and the urea was heated at 550 ºC for 4 h with a rate of 5 ºC/min in a tube furnace under air. The g-C_3N_4 was received after furnace was cooled down to room temperature. The obtained pale-yellow powder was collected and ground with a pestle and mortar for further use.

Synthesis of 1T-MoS_2 nanosheets

1T-MoS_2 was synthesized based on a recent work.[1] In more details, 50 mg of $(NH_4)_6Mo_7O_{24}\cdot 6H_2O$, 80 mg of CH_3CSNH_2, and 10 mL of deionized (DI) water were combined in a 25 mL autoclave and sonicated until they were fully dissolved. Once dissolved, the autoclave was sealed within a hydrothermal reactor and heated at 180°C for 24 h. Further, the reactor was cooled to room temperature and the solution of crude product was transferred to 15 mL conical centrifuge tubes. The crude product was washed and centrifuged three times: once with DI water, once with ethyl alcohol, and once with acetone. The solutions were centrifuged for 10 min at ~4,000 rpm in between each washing step and the supernatant layer was removed after each centrifugation step. After washing, the purified product was dried in a vacuum oven at ~80 °C and ~25 mmHg for 24 h. The dried and purified product was finely ground with a mortar and pestle and stored under ambient conditions.

Synthesis of MoS_2/g-C_3N_4 composite

0.1 g g-C_3N_4 was ground and dispersed into 2 mL distilled water (DI H_2O). Then, 1 mg mL^{-1}, 1mL of 1T-MoS_2 aqueous solution was added dropwise into the g-C_3N_4 solution to produce 1 wt%-MoS_2/g-C_3N_4 under stirring to form a mixture. After stirring for 1 h and ultrasonic dispersion for 2 h, the mixture was dried in an oven at 85 ºC for 24 h to evaporate the solvent. The obtained solid was ground and loaded into a crucible, and then heated at 300 ºC for 2 h with a ramp rate of 5 °C/min under N_2 flow in a tube furnace. Other loading amounts of MoS_2 on g-C_3N_4 were synthesized via the similar procedure. The loading amount of MoS_2 was optimized based on the photocatalytic hydrogen evolution reaction (HER) performance as shown in Table S2.

Photocatalyst Characterizations

Powder X-ray diffraction (XRD) patterns were measured with a Bruker D8 Advance X-ray diffractometer and Rigaku SmartLab X-ray diffractometer using Cu-Kα radiation (λ = 1.5418 Å).

UV-Visible spectra were measured using a Shimadzu UV-2600 with an integrating sphere attachment. Powder samples were mixed with a known amount of non-absorbing $BaSO_4$. The Kubelka-Munk transform was applied to the raw diffuse reflectance data.

F(R) = (1-R)^2/2R

Photoluminescence (PL) measurements were recorded on a PTI Quantamaster QM-400 fluorescence spectrophotometer with an excitation wavelength of 360 nm. All samples were prepared at a concentration of 2.0 mg mL^{-1} in 1 M KOH aqueous solution in a quartz glass cuvette (1 cm path length).

Fourier transform infrared (FTIR) spectra were obtained using a PERKIN ELMER FTIR spectrometer.

Raman spectra were recorded using a Thermo Scientific DXR Raman microscope, employing an Ar-ion laser operating at 532 nm.

X-ray photoelectron spectroscopy (XPS) was collected using a PHI 5600 XPS machine equipped with an Al Kα X-ray beam (1486.7 eV), ran at 250 W, and operated at 14 kV. All binding energies were calibrated by the reference C 1s peak at 284.40 eV. Samples for XPS were prepared through dispersing into acetone (~2 mg mL^{-1}), drop-cast onto carbon fiber paper, and dried.

The electrochemical impedance spectra (EIS) and Mott-Schottky measurements were conducted in an electrochemical cell with a standard three-electrode configuration composed with a Pt mesh as the counter electrode and an Ag/AgCl as the reference electrode. 0.2 M Na_2SO_4 aqueous solution was used as the electrolyte and CHI660E was employed as the electrochemical workstation for electrochemical measurements.

To prepare the working electrode, 4.5 mg as-synthesized sample and 50 μL nafion solution (5 wt%) were ultrasonically dispersed in 1 mL absolute ethanol to form a heterogeneous ink. Then, 150 μL of catalysts ink was uniformly dropped onto a FTO with 1 × 2 cm^2 area and allowed to dry at 60 ºC in an oven for at least one hour.

Scanning transmission electron microscopy (STEM) was performed using the JEOL Grand and high-angle angular dark-filled (HAADF)-STEM images were acquired at a convergence semiangle of 22 mrad and inner and outer collection angles of 83 and 165 mrad, respectively.

Energy dispersive X-ray spectroscopy (EDS) was conducted using JEOL dual EDS detectors and a specific high count analytical TEM holder.

Scanning electron microscopy (SEM) analyses were collected using the FEI Quanta 450 FEG SEM at 20 eV.

Pretreatment of PET powder and PET water bottle

6.3 g PET powder was soaked into 100 mL 2 M KOH solution in a sealed flask for 18 h at 60 °C with stirring (500 rpm). After the hydrolysis reaction, the mixture was separated by centrifuge. The obtained clear aqueous solution was denoted as the PET powder hydrolysate and directly used for further analysis and reactions.

PET water bottle was cut into small pieces with around 0.5 cm × 0.5 cm size, 5 g water bottle pieces were then soaked into 100 mL 2 M KOH solution in a sealed flask for 48 hours at 60 °C with stirring (500 rpm). After centrifuge, the obtained clear solution was denoted as the PET water bottle hydrolysate.

Pretreatment of plastic mixtures

PET + LDPE: 3 g PET and 3 g LDPE

PET + PS: 3 g PET and 3 g PS

PET + LDPE + PS: 2 g PET, 2 g PS, and 2g LDPE

Each plastic mixture was soaked into 100 mL 2 M KOH solution in a sealed flask for 24 h at 60 °C with stirring (500 rpm) for hydrolysis. After hydrolysis, the solid residue and hydrolysate solution were separated by centrifuge. The obtained clear aqueous solution was denoted as the plastic mixture hydrolysate and directly used for further analysis and reactions. After hydrolysis, the products in the hydrolysates were analyzed and quantified by LC-MS (Figure S26) and ^{1}H NMR (Figure S25).

Liquid Chromatography-Mass Spectrometry (LC-MS)

PET waste hydrolysates were analyzed by LC-MS: 100 µL PET powder or PET water bottle hydrolysate was diluted to 10 ml with methanol and then analyzed by LC-MS (Agilent 6530B accurate-MASS Q-tof LC/MS).

Photocatalysis Experiments

Photocatalytic Hydrogen Evolution Reaction (HER)

2 mg photocatalyst (MoS_2/g-C_3N_4) was dispersed into 1.25 mL solution which contains 1 mL solvent (DI water, 1 M KOH, or 2 M KOH) and 0.25 mL electron donor (TEOA), the headspace of reaction cell was measured to be 0.85 mL. Prior to photocatalysis, the mixture was ultrasonicated for 5 min and subsequently purged with N_2 (flow rate, 10 sccm) for 10 min to remove any possible oxidization species.

In photocatalysis experiments, the sample was irradiated by the Xenon lamp equipped with a long-pass (λ > 400 nm) optical filter and a water filter to remove infrared radiation as shown in Figure S13. Lamp light intensity on the reaction cell was calibrated to 1 sun. The head space above the solution was sampled with a gas-tight syringe at different time intervals for products analysis using a Shimadzu GC equipped with a BID detector.

Photocatalytic Oxidation of EG, TPA and PET hydrolysates

0.3 M EG and 0.3 M TPA solution were prepared through dissolving certain amounts of chemicals into 2 M KOH. Subsequently, 2 mg photocatalyst was dispersed into 1.25 mL of 0.3 M EG or 0.3 M TPA solution for photocatalysis under same conditions as that for photocatalytic HER.

For PET waste, 1.25 mL commercial PET powder hydrolysate or PET water bottle hydrolysate were directly applied to the photocatalysis system as well.

Photocatalytic oxidation of PET plastic wastes under Sun Light

2 mg photocatalysts were directly dispersed into 1.25 mL commercial PET powder hydrolysate or PET water bottle hydrolysate, respectively. The mixture was put under sunlight for 2 hours with stirring. Sun light intensity was tested by the light meter.

Gas products analysis

The composition and distribution of gaseous products were analyzed by gas chromatography (SHIMADZU, GC-2010) equipped with a BID detector. Gaseous product yield was calculated based on calibration curves shown in Figure S17.

Calculation equations:

The gas products yield was calculated based on the following equation:

$$Yield(mol/mg) = \frac{1 * (c * V)}{R * (T + 273) * m}$$

c: the concentration of gas products from GC

V: the volume of headspace of reaction vial

R: gas constant

T: reaction temperature

m: mass of catalyst used for reaction.

Liquid products identification and quantitative analysis

After photocatalysis, the mixture was separated by centrifuge. The clear solution was analyzed and identified by ^{1}H NMR spectroscopy with DMSO as internal standard (ISD). ^{1}H NMR spectra were collected on a 400 MHz Varian spectrometer. The NMR samples with DMSO was prepared through mixing 486 μL solution after photocatalysis, 54 μL DMSO and 60 μL D_2O. The NMR samples without ISD was prepared by mixing 540 μL solution after photocatalysis and 60 μL D_2O. The water peak in all ^{1}H NMR spectra was removed from 4 to 6 ppm via adding a break.

Liquid product yield was calculated according to the corresponding standard chemicals calibration curves. Calibration curves were obtained through plotting standard chemicals concentration with ^{1}H NMR peak integration area as shown in Figure S18.

Table S1. Annealed and unannealed $MoS_2/g\text{-}C_3N_4$. Photocatalytic conditions (unless stated otherwise below): 2 mg $MoS_2/g\text{-}C_3N_4$, 1.00 mL H_2O, 0.25 mL TEOA into a 2.1 mL vial, the mixture was ultraosonicated for 5 min and purged with N_2 for 10 min prior to photocatalysis. Simulated solar light condition: Xe lamp, 1 sun intensity.

Sample description	Synthesis condition	Time (min)	Yield (μmol g^{-1})	Rate (μmol g^{-1} h^{-1})
0.5 wt% $MoS_2/g\text{-}C_3N_4$	without heating	20	0.33	1.00
		40	0.39	0.57
		60	0.70	0.70
		80	0.49	0.36
		100	0.55	0.33
		120	1.01	0.51
	at 300 °C for 2 hours	20	8.72	26.17
		40	17.37	26.05
		60	23.16	23.16
		80	26.02	19.51
		100	28.80	17.28
		120	31.08	15.54

Table S2. Catalysts loading amounts of $MoS_2/g\text{-}C_3N_4$. Photocatalytic conditions (unless stated otherwise below): 2 mg $MoS_2/g\text{-}C_3N_4$, 1.00 mL H_2O, 0.25 mL TEOA into 2.1 mL vial, the mixture was ultraosonicated for 5 min and purged with N_2 for 10 min prior to photocatalysis. Simulated solar light condition: Xe lamp, 1 sun intensity.

Description	MoS_2 loading (wt%)	Time (min)	H_2 Yield (μmol g^{-1})	Rate (μmol g^{-1} h^{-1})
MoS_2 loading amount	0.5	20	8.72	26.17
		40	17.37	26.05
		60	23.16	23.16
		80	26.02	19.51
		100	28.80	17.28
		120	31.08	15.54
	1	20	8.61	25.83
		40	17.55	26.33
		60	26.19	26.19
		80	29.72	22.29
		100	32.42	19.45
		120	35.73	17.87
	2	20	4.11	12.32
		40	5.72	8.58
		60	9.27	9.27
		80	10.62	7.97
		100	12.60	7.56
		120	12.53	6.26

Note: 1 wt%-$MoS_2/g\text{-}C_3N_4$ gives the optimal photocatalytica H_2 performance. Thus, 1 wt%-$MoS_2/g\text{-}C_3N_4$ was referred as $MoS_2/g\text{-}C_3N_4$ if no further notifications.

Table S3. Substrate photooxidation control experiments. Photocatalysis conditions: 2.0 mg catalyst dispersed into 1.25 mL substrate solution (in 2 M KOH) for 2 hours. Simulated solar light condition: Xe lamp, 1 sun intensity.

Sample	Substrate	H_2 yield (μmol g^{-1})	CH_4 yield (μmol g^{-1})	CO yield (μmol g^{-1})
Air	/	0.21	0	0.55
No catalysts	PET powder hydrolysate	0.40	0.30	0.40
No light	PET powder hydrolysate	0.58	0.49	0.04
g-C_3N_4	PET powder hydrolysate	1.26	1.72	3.29
MoS_2/g-C_3N_4	PET powder hydrolysate	3.93	1.49	1.41
MoS_2/g-C_3N_4	0.3 M terephthalic acid	0.47	0.47	0.52
MoS_2/g-C_3N_4	0.3 M ethylene glycol	8.17	3.63	0.74
MoS_2/g-C_3N_4	PET water bottle hydrolysate	1.05	1.56	2.06

Table S4. H_2 yield with lactic acid and glycolic acid as substrates. Photocatalysis conditions: 2.0 mg catalyst dispersed into 1.25 mL substrate solution (in 2 M KOH) for 2 hours. Simulated solar light condition: Xe lamp, 1 sun intensity.

Sample	Substrate	Time (h)	H_2 yield (μmol g^{-1})
MoS_2/ g-C_3N_4	0.06 M Lactic acid	2	3.64
MoS_2/ g-C_3N_4	0.3 M Lactic acid	2	5.29
MoS_2/ g-C_3N_4	0.3 M Glycolic acid	2	9.67

Table S5. Quantification of liquid products of PET powder hydrolysate and PET water bottle hydrolysate after 2 hours photocatalysis under nature conditions.

		Substrates photocatalysis	
		PET powder hydrolysate	PET water bottle hydrolysate
Products quantity (nmol)	Formate	120.77	/
	Glycolate	53.86	/
	Acetate	135.52	46.85

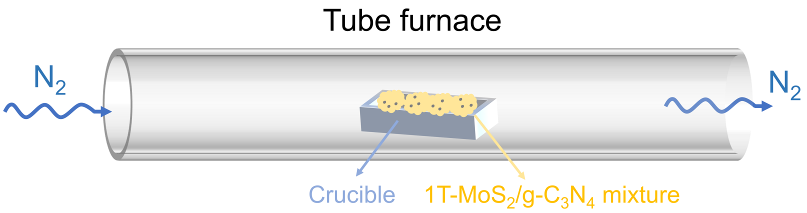

Figure S1. Schematic illustration of the synthesis process of MoS_2/g-C_3N_4.

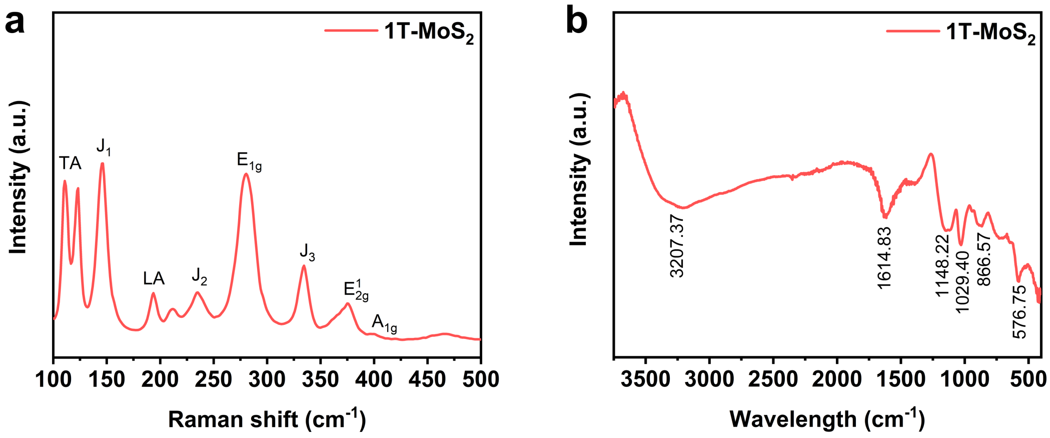

Figure S2. Raman spectrum (a) and FTIR (b) of the as synthesized 1T-MoS_2.

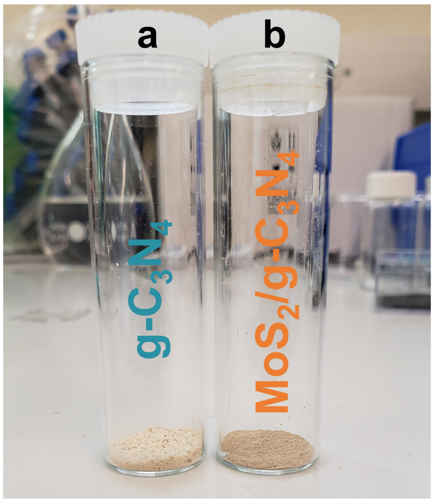

Figure S3. Photography of g-C_3N_4 (a) and MoS_2/g-C_3N_4 (b).

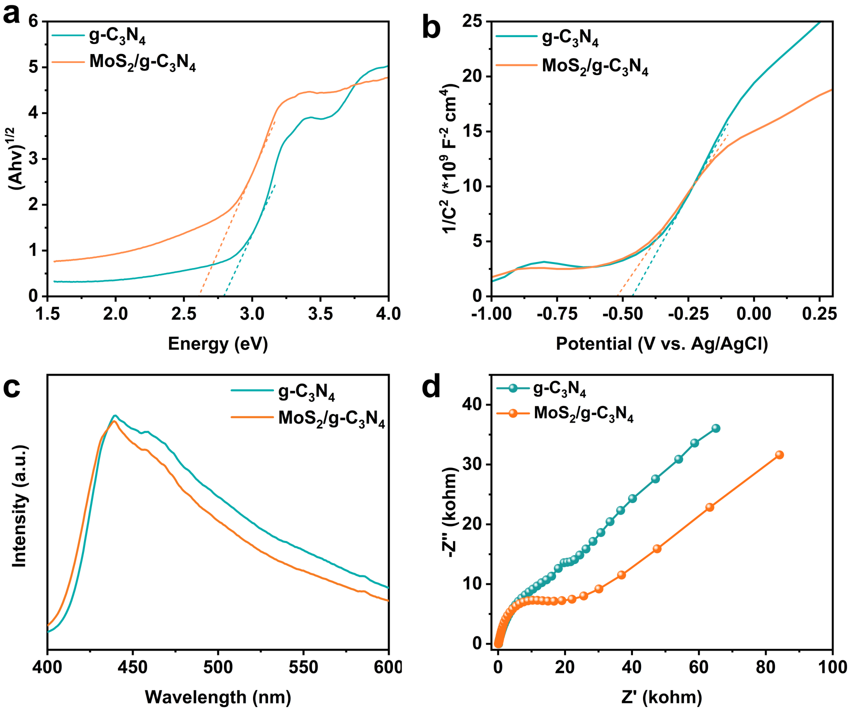

Figure S4. (a) Tauc plot derived from corresponding diffuse-reflectance UV-vis spectra. (b) Mott-Schottky (M-S) curves. M-S curves were acquired at a frequency of 1.5 kHz from -1.0 V to 0.3 V (vs. Ag/AgCl) (c) Photoluminescence (PL) spectra of g-C_3N_4 and MoS_2/g-C_3N_4 under the excitation wavelength of 360 nm and (d) EIS Nyquist plot of g-C_3N_4 and MoS_2/g-C_3N_4. EIS study was carried out under operation condition at -0.4 V vs. Ag/AgCl. Amplitude was set to be 5 mV and frequency was set from 0.01 Hz to 100 kHz.

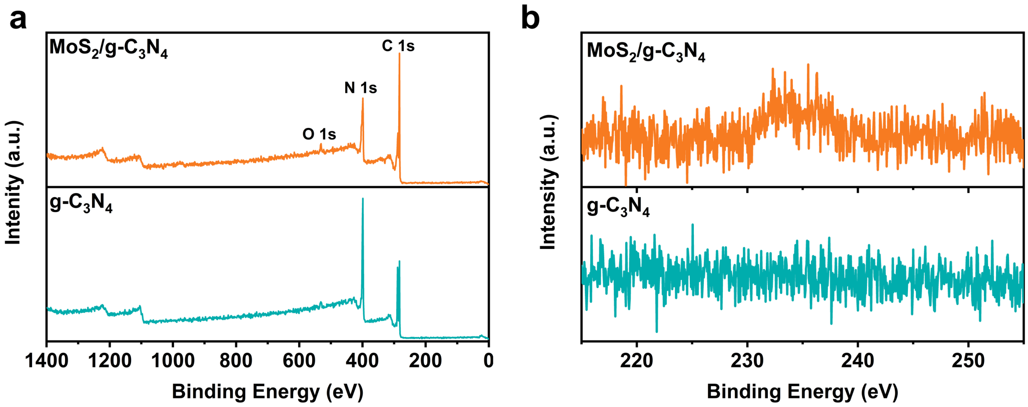

Figure S5. X-ray photoelectron spectroscopy (XPS) of g-C_3N_4 and MoS_2/g-C_3N_4. (a) survey spectra, (b) Mo 3d spectra.

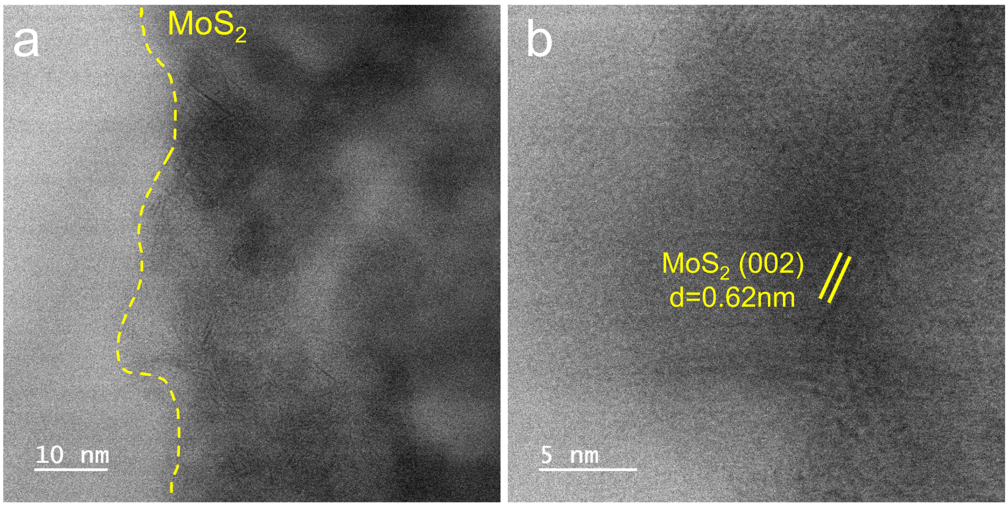

Figure S6. STEM images of MoS_2/g-C_3N_4. **Figure S6b** shows the lattice distance of 0.620 nm, corresponding to (002) plane of MoS_2.

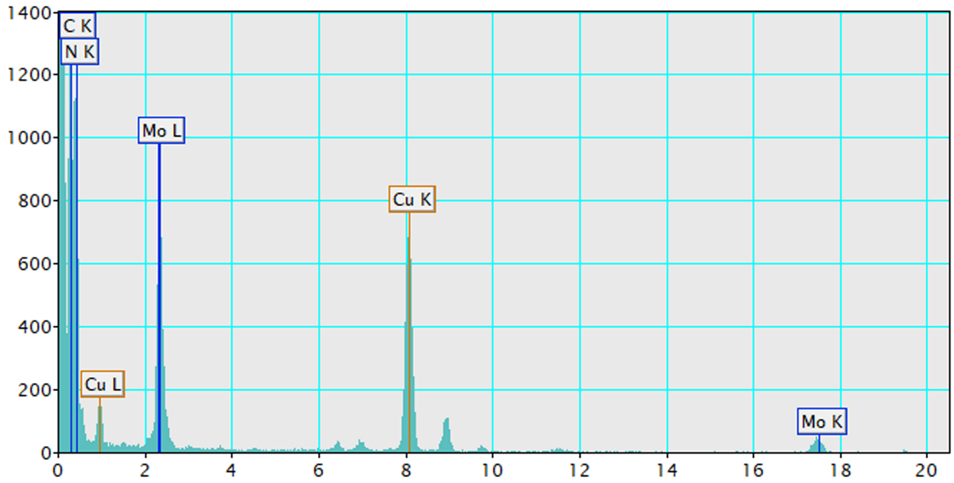

Figure S7. The energy dispersive intensity profiles of existing elements in the MoS_2/g-C_3N_4 sample. C, N, and Mo were clearly observed. Herein, the Cu peak is derived from the copper STEM grinds.

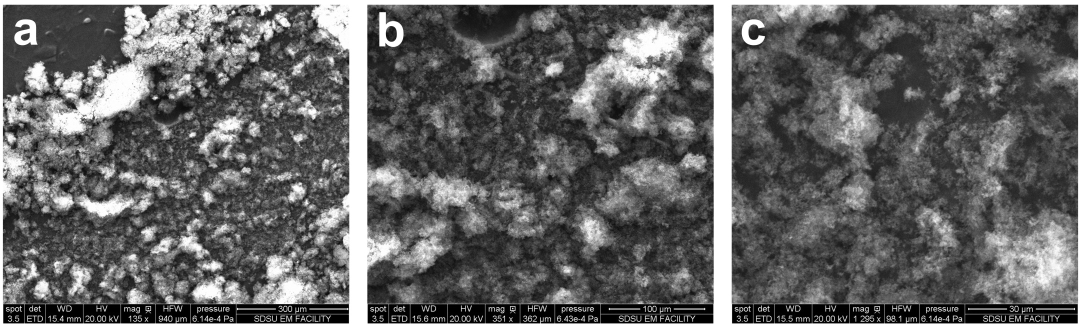

Figure S8. (a-c) SEM images of g-C_3N_4.

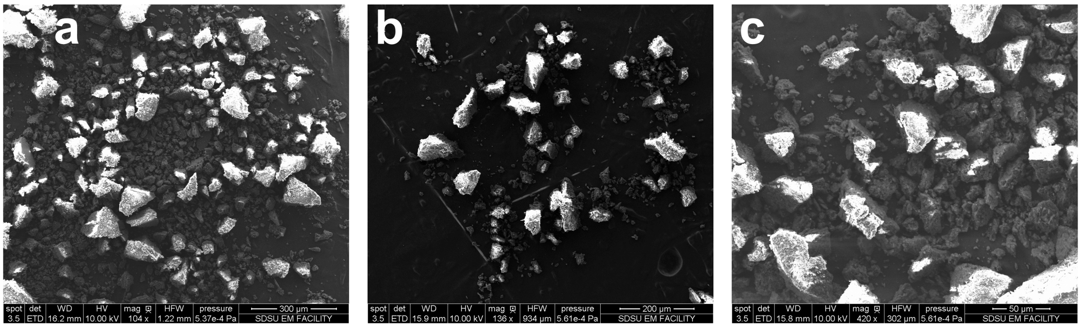

Figure S9. (a-c) SEM images of g-C_3N_4/MoS_2.

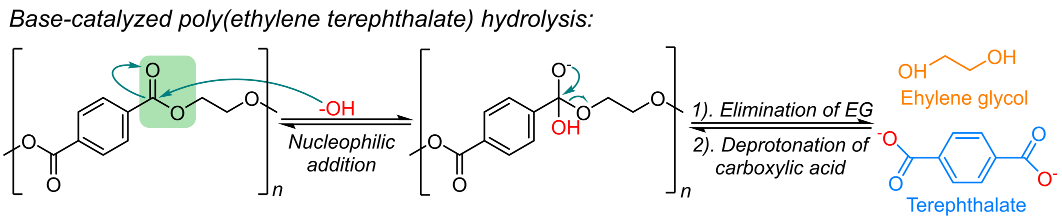

Figure S10. Base-catalyzed poly(ethylene terephthalate) polymer hydrolysis mechanism.

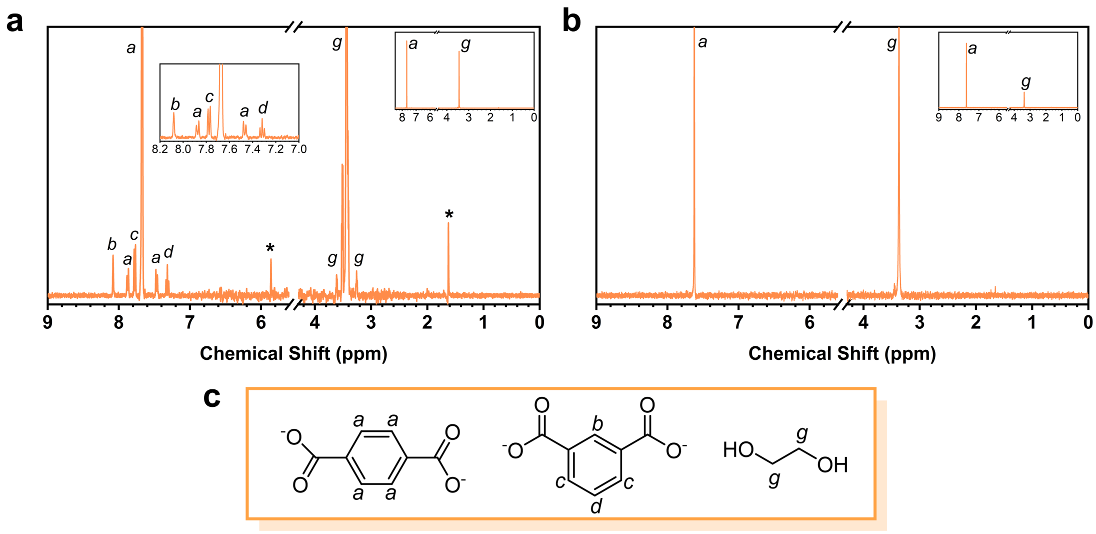

Figure S11. ^{1}H NMR spectra of PET powder hydrolysate and PET water bottle hydrolysate (a and b, respectively) and (c) corresponding peak assignments and chemical structures.

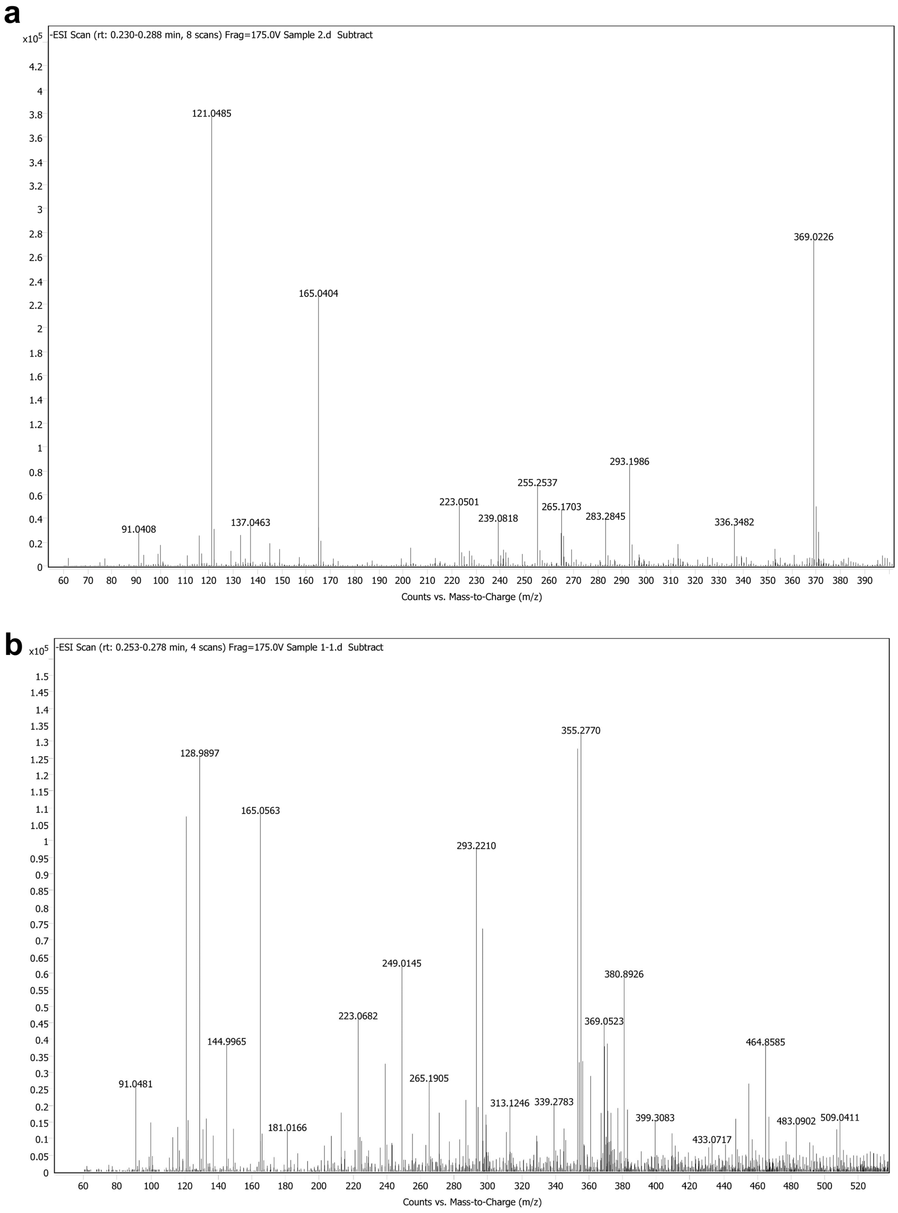

Figure S12. Liquid chromatography-mass spectroscopy (LC-MS, negative ion mode) of (a) PET powder hydrolysate and (b) PET water bottle hydrolysate after pretreatment.

PET power and PET water bottle hydrolysates were diluted 100 times with methanol for the LC-MS analysis. For PET powder hydrolysate (**Figure S12a**), the peak at 165 m/z is assigned to terephthalate and the peak at 121 m/z corresponds to the formation of benzoate (a fragment of terephthalate). The peaks at 369 m/z, 293 m/z might be derived from oligomer fragments.

For PET water bottle hydrolysate (**Figure S12b**), the major peaks are similar as PET powder hydrolysate. Compared to PET powder hydrolysate, more minor peaks, which might be from the impurities of commercial PET water bottle, were observed. In addition, the terephthalate peak was observed at 165 m/z, which matches the ^{1}H NMR data.

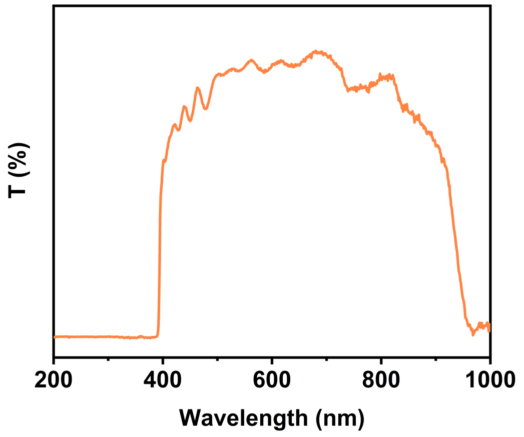

Figure S13. The output spectra of the Xenon lamp used in this study with a 400 nm long pass filter and water filter.

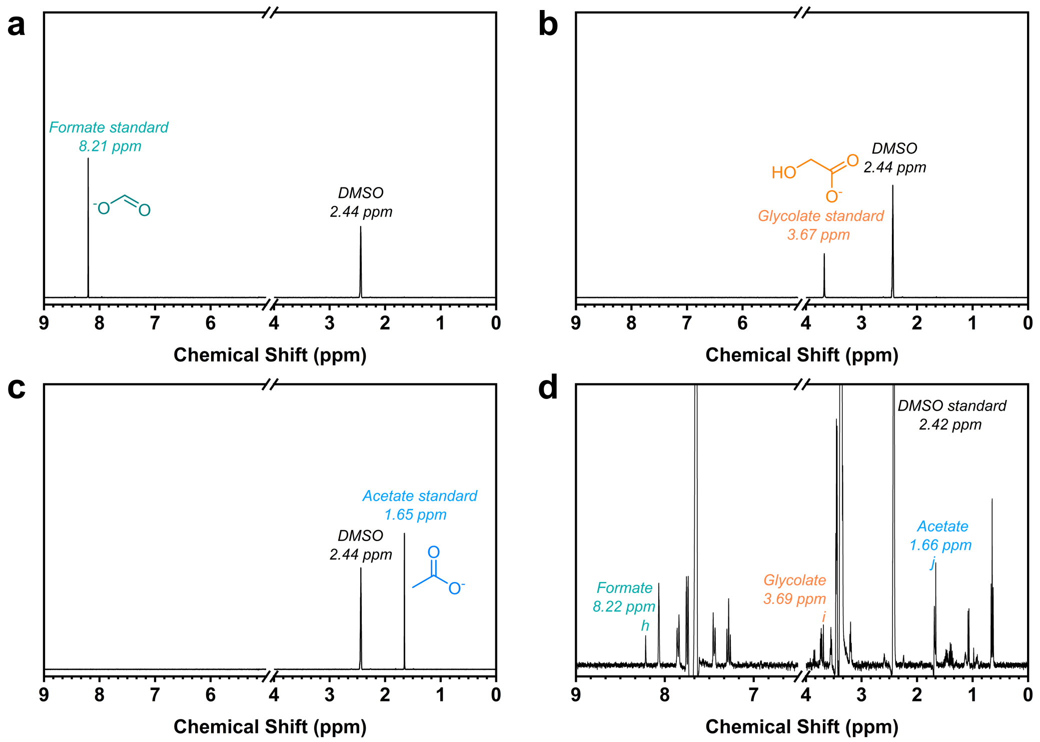

Figure S14. ^{1}H NMR spectra of standard chemicals: (a) formate (50 mM), (b) glycolate (10 mM), (c) acetate (10 mM) in 2 M KOH. And (d) PET hydrolysate after a 42 hours photocatalysis with the DMSO as an internal standard. Photocatalysis conditions: 2 mg MoS_2/g-C_3N_4, 1.25 mL substate, 1 sun of Xe lamp light intensity.

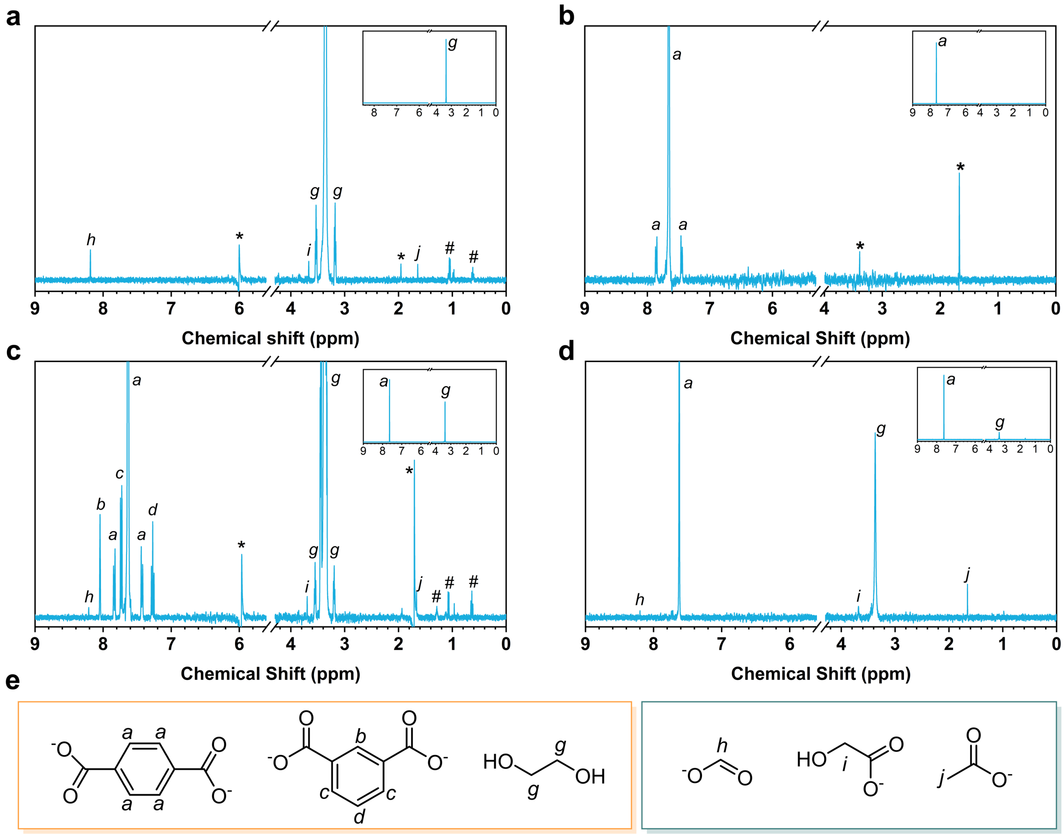

Figure S15. ^{1}H NMR spectra of (a) ethylene glycol (0.3 M in 2 M KOH), (b) terephthalic acid (0.3 M in 2 M KOH), (c) PET powder hydrolysate, (d) PET water bottle hydrolysate after 2 hours photocatalysis, and (e) corresponding peak assignments and chemical structures. Photocatalysis conditions: 2 mg MoS_2/g-C_3N_4, 1.25 mL substate, 1 sun of Xe lamp light intensity.

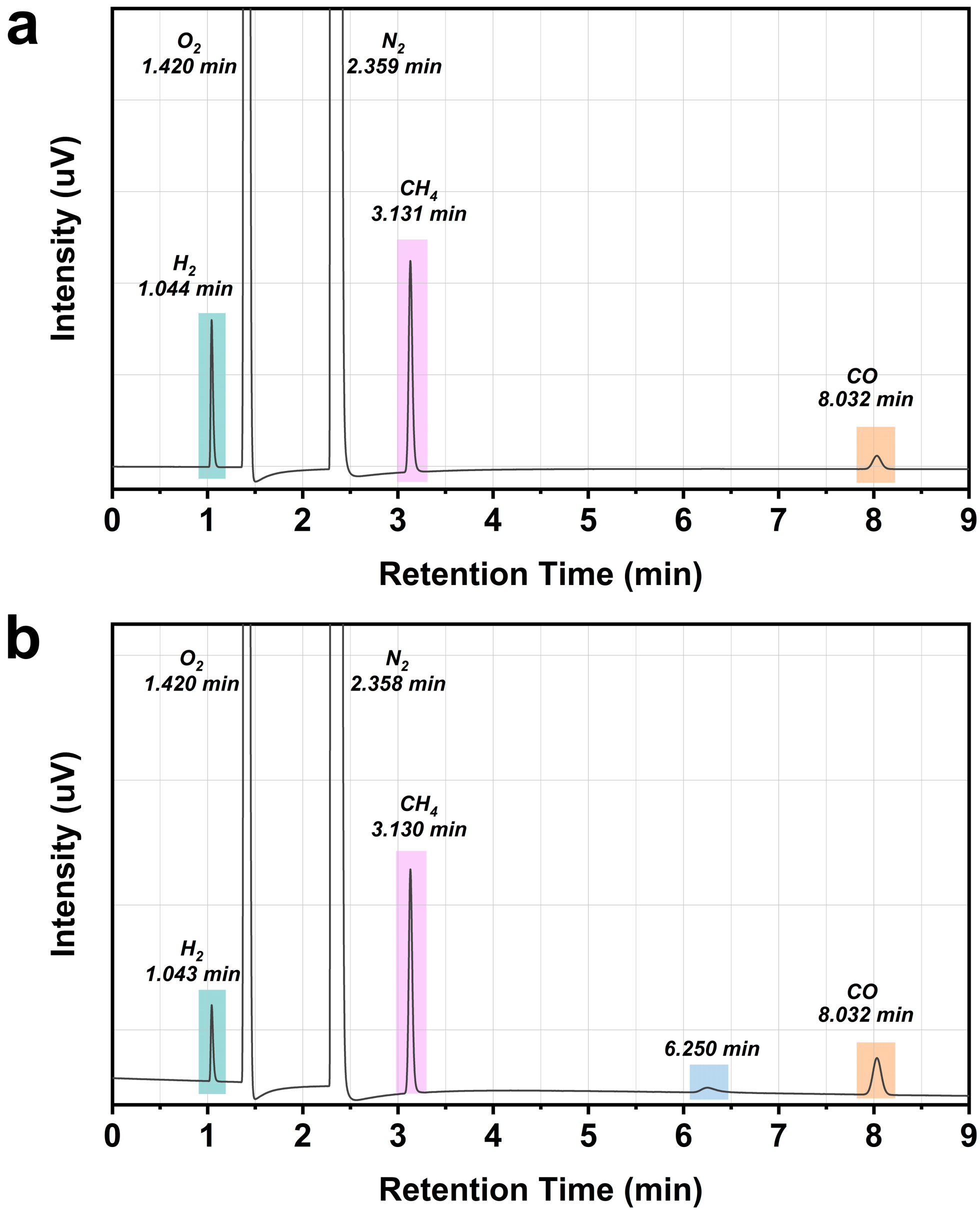

Figure S16. GC curves of gas product mixtures after 42 hours photocatalysis for (a) PET powder hydrolysate and (b) PET water bottle hydrolysate.

For PET water bottle hydrolysate, after 42 hours of photocatalysis, the gas product peak at the retention time of 6.250 min might be originated from the impurities of PET water bottle.

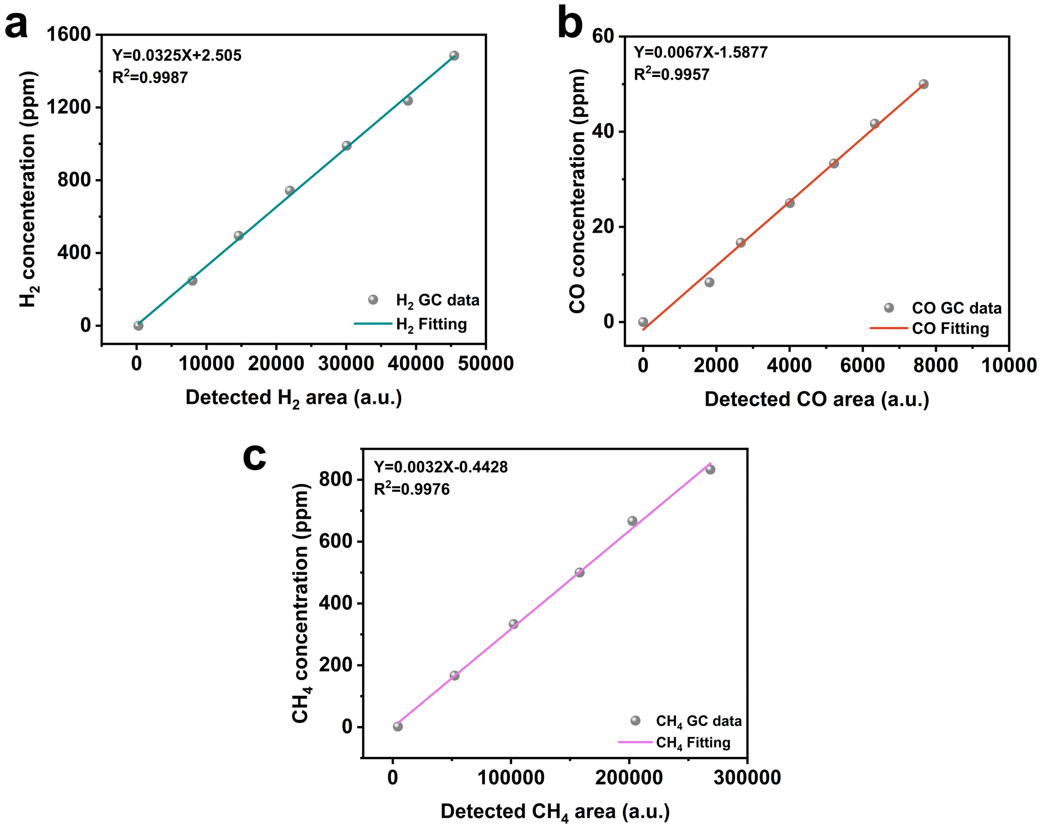

Figure S17. Gas products calibration curves with GC (a) hydrogen (b) carbon monoxide (c) methane.

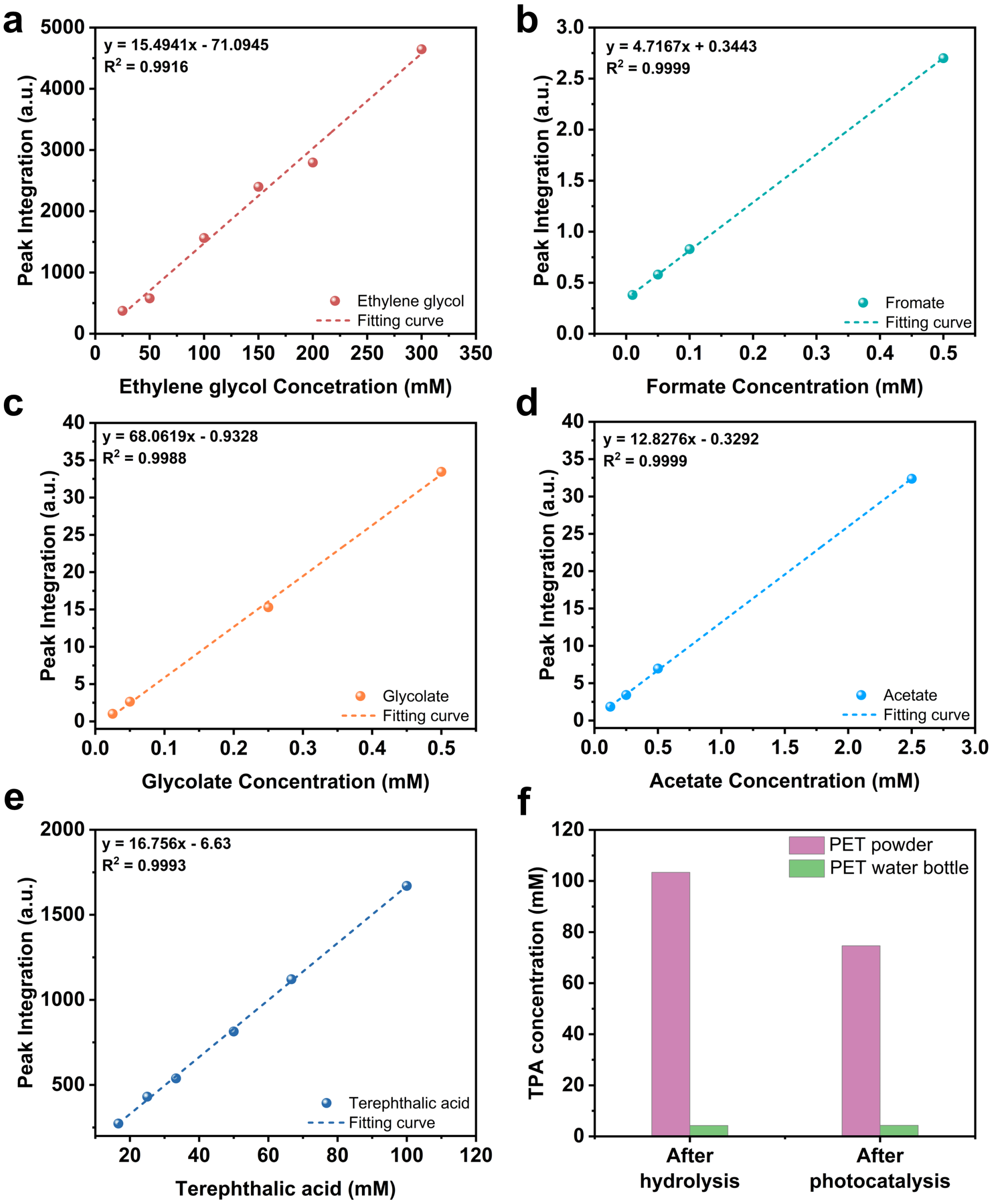

Figure S18. Quantitative calibration curves for the liquid products in 1 M KOH. (a) ethylene glycol, (b) formate, (c) glycolate, (d) acetate, (e) terephthalate, (f) terephthalate concentration of PET powder and PET water bottle after hydrolysis and after 42 hours photocatalysis, respectively.

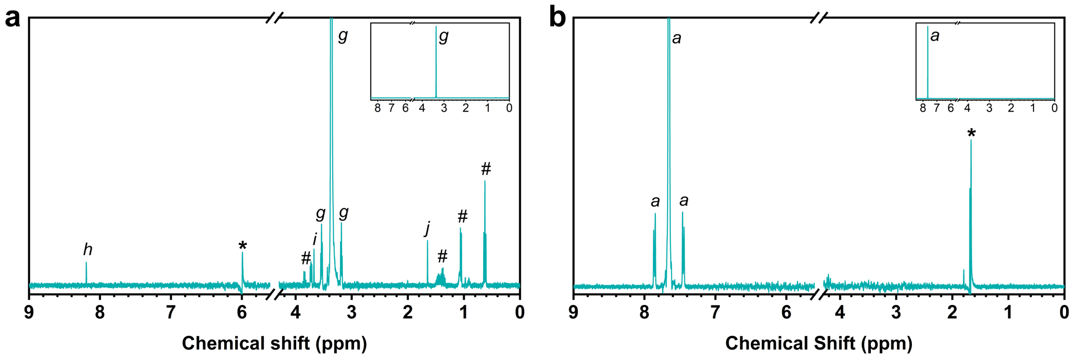

Figure S19. ^1H NMR spectra of (a) ethylene glycol (0.3 M in 2 M KOH), (b) terephthalic acid (0.3 M in 2 M KOH) after 42 hours photocatalysis. (#) refers to unidentified products, those products may be produced from radical coupling reactions.[2] Photocatalysis conditions: 2 mg MoS_2/g-C_3N_4, 1.25 mL substate, 1 sun of Xenon lamp light intensity.

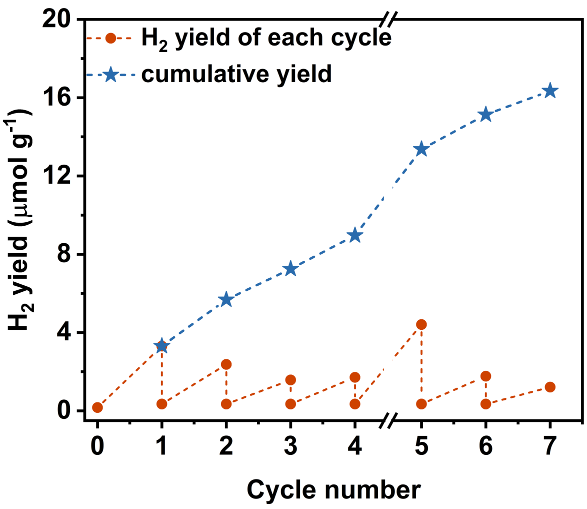

Figure S20. The cycled and cumulative H_2 evolution yield from photocatalytic upcycling of PET powder hydrolysate for successive seven 2-hour cycles. After reaction for 2 h, the photoreactor was purged with N_2 for 5 min. The time gap between cycle-4 and cycle-5 is around 12 hours. Photocatalysis conditions: 2 mg MoS_2/g-C_3N_4, 1.25 mL PET hydrolysate, 1 sun of Xenon lamp light intensity.

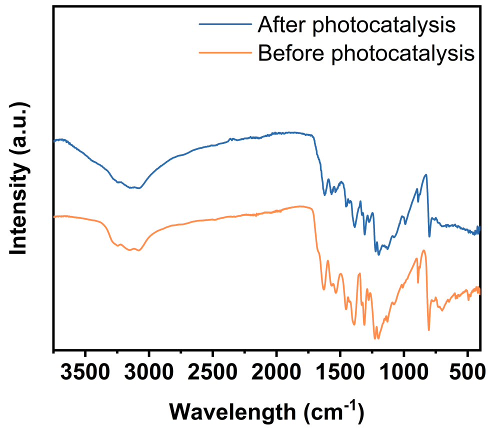

Figure S21. FTIR spectra of $MoS_2/g\text{-}C_3N_4$ before and after photocatalysis. Photocatalysis conditions: 4 mg $MoS_2/g\text{-}C_3N_4$, 2.50 mL PET powder hydrolysate, 1 sun of Xenon lamp light intensity, 24 hours. After photocatalysis, the photocatalyst was separated by centrifuge and dried for FTIR measurements.

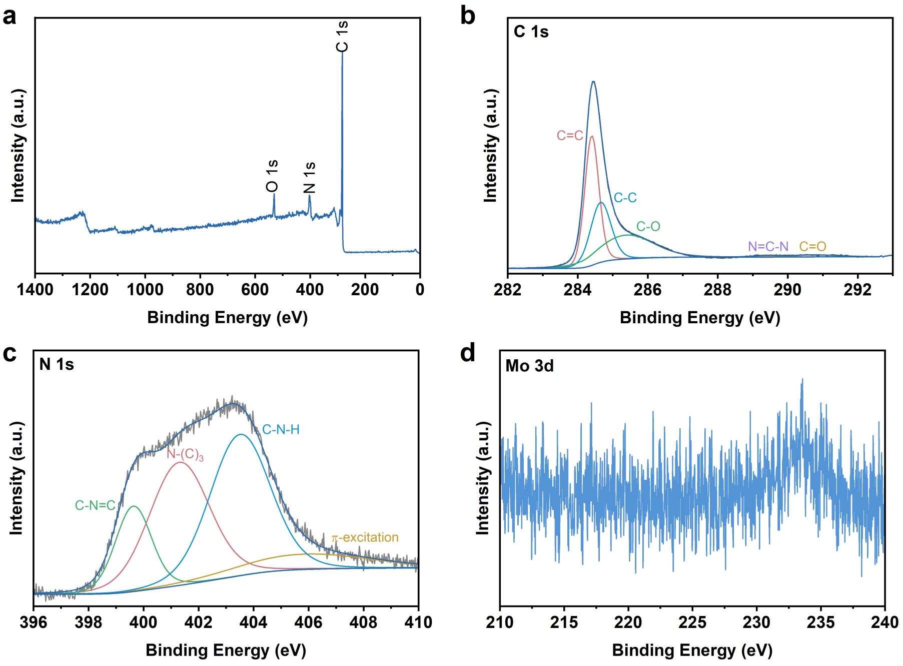

Figure S22. XPS survey spectrum of $MoS_2/g\text{-}C_3N_4$ (a) and the corresponding C 1s (b), N 1s (c), Mo 3d (d) spectra after photocatalysis. Photocatalysis conditions: 4 mg $MoS_2/g\text{-}C_3N_4$, 2.50 mL PET powder hydrolysate, 1 sun of Xe lamp light intensity, 24 hours. After photocatalysis, the photocatalyst was separated by centrifuge and dried for XPS analysis.

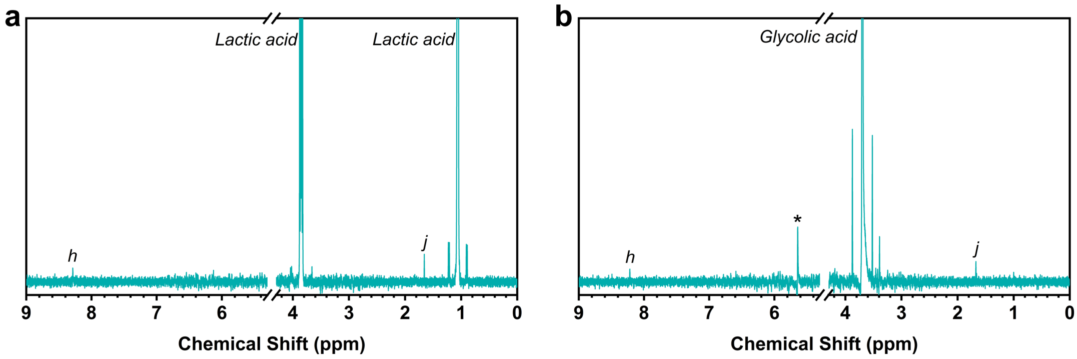

Figure S23. ^{1}H NMR spectra of (a) lactic acid (0.06 M in 2 M KOH), (b) glycolic acid (0.3 M in 2 M KOH) after 2 hours photocatalysis.

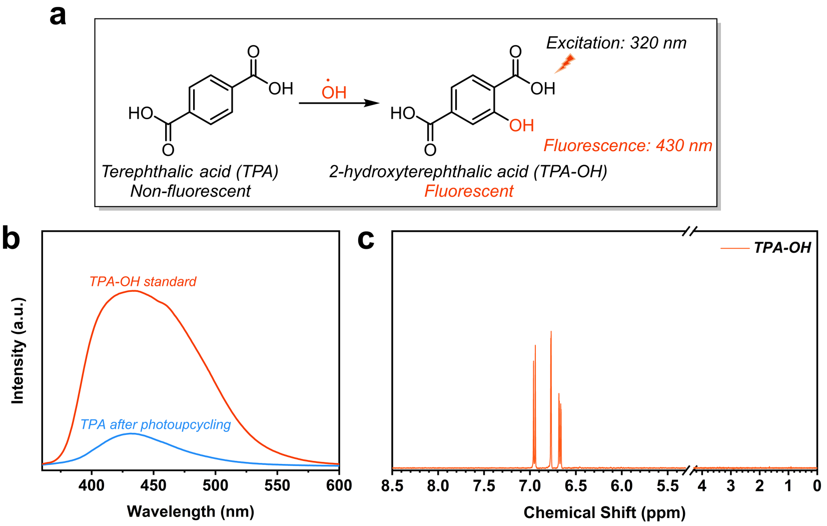

Figure S24. (a) Reaction between the formed •OH and terephthalic acid to produce fluorescent 2-hydroxyterephthalic acid (TPA-OH), (b) Fluorescence spectra of TPA-OH (1.5×10^{-4} M in 2 M KOH) standard and terephthalic acid (0.3 M in 2 M KOH) after 42 hours photocatalysis, (c) ^{1}H NMR spectrum of the TPA-OH standard.

Photocatalytic upcycling of plastic mixtures

To evaluate the photocatalysis performance of $MoS_2/g\text{-}C_3N_4$ in a mixed plastic waste, three types of plastic mixture were prepared.

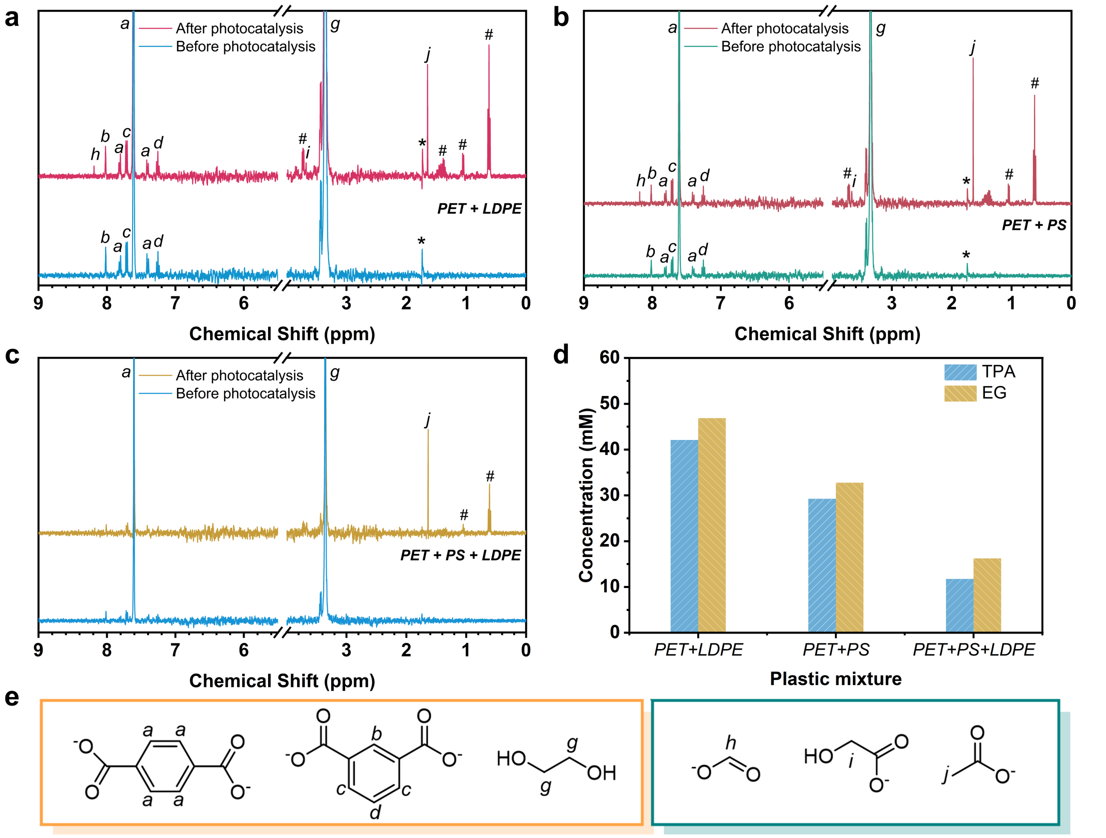

Figure S25. ^{1}H NMR spectra of plastic mixtures before and after 42 hours of photocatalysis. (a) PET + LDPE, (b) PET + PS, and (c) PET + PS + LDPE. (d) TPA and EG concentration in different plastic mixture hydrolysates after 24 hours of hydrolysis in 2 M KOH at 60 °C. (e) corresponding peak assignments and chemical structures. (*) are seen from control experiments. (#) refers to unidentified products. Photocatalysis conditions: 2 mg $MoS_2/g\text{-}C_3N_4$, 1.25 mL plastic mixture hydrolysate, 1 sun of Xenon lamp intensity.

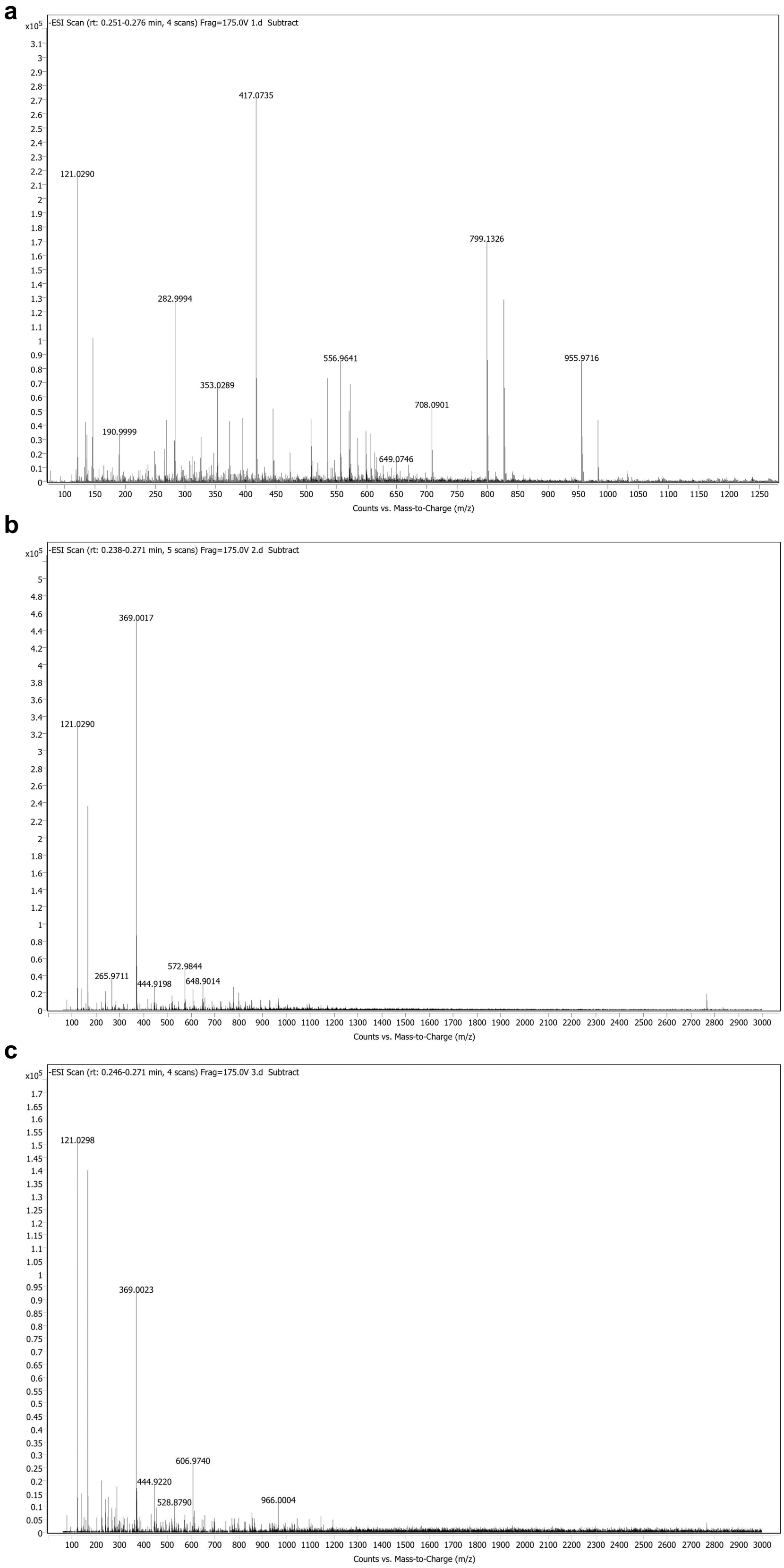

Figure S26. Liquid chromatography-mass spectroscopy (LC-MS, negative ion mode) of plastic mixture hydrolysates after pretreatment (a) PET + LDPE, (b) PET + PS, and (c) PET + PS + LDPE.

Reference

(1) Younan, S. M.; Li, Z.; Yan, X.; He, D.; Hu, W.; Demetrashvili, N.; Trulson, G.; Washington, A.; Xiao, X.; Pan, X.; Huang, J.; Gu, J., Zinc Single Atom Confinement Effects on Catalysis in 1T-Phase Molybdenum Disulfide. *ACS Nano* **2023,** *17*, 1414-1426.

(2) Zheng, X.; Wei, L.; Zhang, Z.; Jiang, Q.; Wei, Y.; Xie, B.; Wei, M., Research on photocatalytic H_2 production from acetic acid solution by Pt/TiO_2 nanoparticles under UV irradiation. *Int. J. Hydrogen Energy* **2009,** *34*, 9033-9041.
